# NGS Approaches in Clinical Diagnostics: From Workflow to Disease-Specific Applications

**DOI:** 10.3390/ijms26199597

**Published:** 2025-10-01

**Authors:** Desiree Brancato, Simone Treccarichi, Francesca Bruno, Elvira Coniglio, Mirella Vinci, Salvatore Saccone, Francesco Calì, Concetta Federico

**Affiliations:** 1 Department of Biological, Geological and Environmental Sciences, University of Catania, 95124 Catania, Italy; desiree.brancato@phd.unict.it (D.B.); elvira.coniglio@phd.unict.it (E.C.); concetta.federico@unict.it (C.F.); 2 Oasi Research Institute-IRCCS, 94018 Troina, Italy; streccarichi@oasi.en.it (S.T.); mvinci@oasi.en.it (M.V.); cali@oasi.en.it (F.C.); 3 Department of Medicine and Surgery, Kore University of Enna, 94100 Enna, Italy; francesca.bruno@unikore.it

**Keywords:** targeted NGS panels, whole exome sequencing, whole genome sequencing, clinical genomics, cardiomyopathies, connective tissue disorders, genetic diagnostics, variant interpretation, target enrichment, variant classification, molecular workflow

## Abstract

Next-Generation Sequencing (NGS) techniques have become a cornerstone of molecular diagnostics, enabling high-throughput, parallel analysis of multiple disease-associated genes. Their targeted design allows streamlined interpretation and optimised diagnostic yield, especially in disorders with known genetic heterogeneity. In this review, we provide a comprehensive overview of the clinical application of NGS techniques—targeted gene panels, whole exome sequencing (WES) and whole genome sequencing (WGS)—detailing the methodological workflow and the critical steps involved in their implementation. Particular emphasis is placed on the genes identified through NGS that are implicated in neurodevelopmental, neurodegenerative, psychiatric, neuromuscular, cardiovascular, and metabolic disorders. We also compare the advantages and limitations of panel-based diagnostics versus WES and WGS, and discuss future directions, including the integration of long-read sequencing technologies into multidisciplinary clinical practice. Finally, we consider how these advances may ultimately bridge biomedical research and clinical practise to improve the diagnosis and management of multifactorial diseases.

## 1. Introduction

The advent of Next-Generation Sequencing (NGS) technologies has profoundly transformed the landscape of clinical genetics, enabling the simultaneous analysis of multiple genes with unprecedented speed, accuracy, and cost-effectiveness [1,2,3]. Among the various NGS-based approaches, targeted gene panels represent a pragmatic and widely adopted solution for the molecular diagnosis of rare and complex genetic disorders. These panels focus on a predefined set of genes known to be associated with specific phenotypic traits or disease categories, offering high analytical sensitivity through deep coverage of the targeted sequences [4].

In clinical practice, NGS gene panels are particularly valuable when the patient’s phenotype points to a well-characterised group of conditions with known genetic heterogeneity, such as cardiomyopathies, connective tissue disorders, inherited retinal dystrophies, immunodeficiencies, inherited cancer predispositions and solid tumour management [5,6,7,8,9]. Their application reflects a broader shift towards functional and structural genomic diagnostics, as underscored by recent advances in clinical cytogenomics and genome architecture [10]. Furthermore, gene panels are routinely updated to incorporate new disease-associated genes as they are validated, making them highly adaptable tools in precision medicine [11].

On the other hand, the introduction of whole-exome sequencing (WES) as a routine tool in clinical genetics has significantly reduced reliance on targeted gene panels, particularly in the context of multifactorial diseases with poorly defined genetic etiologies. WES is now commonly applied in the diagnostic evaluation of neurodevelopmental disorders, such as autism spectrum disorder (ASD), intellectual disability (ID), and various psychiatric conditions [12,13,14,15]. Typically performed on the proband and both parents (trio analysis), WES facilitates the identification of causative or potentially contributory variants related to the patient’s condition. Moreover, WES enables the detection of variants of uncertain significance (VUS) in genes not previously linked to the patient’s phenotype, thus supporting the discovery of new candidate genes [16,17].

In this review, we aim to provide a comprehensive yet focused overview of the role of NGS approaches in clinical diagnostics. We describe the technical and interpretative workflow underpinning their implementation—from gene selection and target enrichment to sequencing, variant calling, filtering, and clinical interpretation [18,19]. We then illustrate key diagnostic applications across diverse clinical domains, highlighting representative disorders and use cases. Particular emphasis is given to the NGS techniques employed in the diagnostic evaluation of monogenic, neurodevelopmental, psychiatric, neuromuscular, connective tissue, cardiovascular, inherited cancer, neurodegenerative, and metabolic disorders.

Finally, we provide a comprehensive comparison between targeted gene panels and WES, highlighting the advantages and the current limitations of both approaches. We discuss future directions, including the integration of broader genomic techniques and emerging bioinformatic strategies [20,21,22]. This perspective aligns with the evolving understanding of gene origins, dynamics, and functional implications in eukaryotes [23], which inform the design and interpretation of modern diagnostic panels. Overall, we aim to guide clinicians, geneticists, and laboratory professionals in the informed use of NGS as a powerful diagnostic resource in modern genomic medicine, by illustrating practical workflows, clinical applications, and current challenges in interpretation.

## 2. Principles and Diagnostic Workflow of NGS Gene Panels and WES

### 2.1. Clinical Diagnosis in the NGS Era

NGS-based gene panels represent a targeted, efficient, and scalable approach to genetic diagnostics, particularly suitable for disorders with known or suspected genetic heterogeneity. Unlike broader techniques such as whole-exome sequencing (WES) or whole-genome sequencing (WGS), gene panels focus on a predefined set of genes associated with specific clinical phenotypes, thereby increasing diagnostic yield, reducing incidental findings, and lowering the complexity of data interpretation [1,9]. These panels allow the simultaneous sequencing of multiple disease-associated genes, making them a powerful tool for diagnosing genetic conditions. This targeted approach is especially effective in identifying the molecular causes of a wide range of disorders, from rare monogenic diseases to more complex—but well-defined—multifactorial conditions.

On the other hand, WES offers a broader and more comprehensive approach to genetic diagnostics by capturing and analyzing the protein-coding regions (about 2% of the entire genome), which harbor the majority of known disease-causing variants. Unlike targeted gene panels, WES is not limited to a predefined gene set, making it particularly valuable in cases where the genetic basis of a disease is unclear or heterogeneous [13,15,24]. This approach is especially powerful in the investigation of multifactorial disorders—such as neurodevelopmental, psychiatric, and metabolic conditions—where multiple genetic and environmental factors may contribute to disease onset and progression. By enabling the identification of rare or novel variants across the exome, WES supports both diagnostic accuracy and gene discovery [25,26,27]. Additionally, when applied in a trio-based analysis (proband and parents), WES facilitates the detection of de novo, inherited, or compound heterozygous variants, providing critical insights into the genetic architecture of complex diseases. Despite its higher analytical burden and potential for incidental findings, WES remains a cornerstone in modern clinical genomics, especially when standard diagnostic approaches fail to yield conclusive results.

Despite its more limited use due to higher costs and the complexity of data analysis, whole-genome sequencing (WGS) offers the most comprehensive view of the human genome by capturing both coding and non-coding regions [28,29]. Unlike targeted panels or WES, WGS allows for the detection of a broader range of variant types, including structural variants, copy number variations (CNVs), and deep intronic mutations. Although its higher cost, data volume, and complexity of interpretation remain challenges, WGS is particularly valuable in unresolved cases and multifactorial diseases—such as attention deficit hyperactivity disorder (ADHD)—where conventional approaches fail to identify a genetic cause [30].

**Figure 1:**
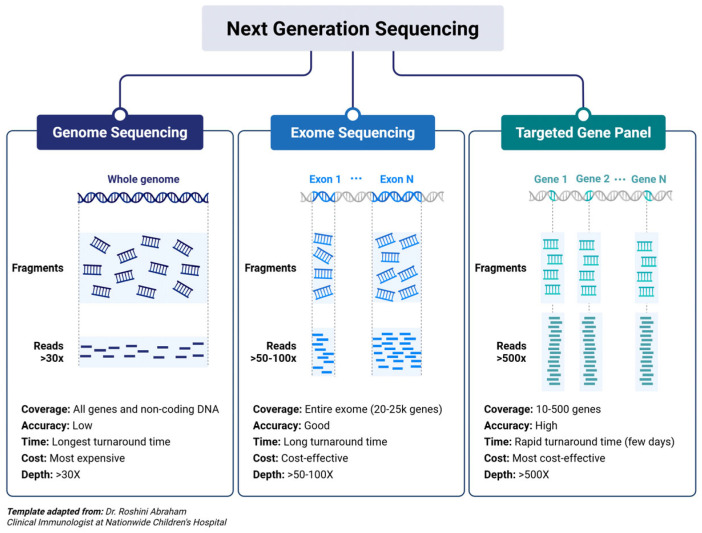
Overview of Next-Generation Sequencing (NGS) approaches: Genome Sequencing, Exome Sequencing, and Targeted Gene Panels. The figure illustrates the key differences among NGS strategies in terms of genomic coverage, sequencing depth, accuracy, turnaround time, and cost. This figure was created in BioRender. Abraham, R. (2025). https://app.biorender.com/profile/template/details/t-61769d43516f2200a7e2d064-comparison-of-next-generation-sequencing-techniques.

A comprehensive comparison of the three approaches is provided in Table 1 and in Figure 1.

**Figure 2:**
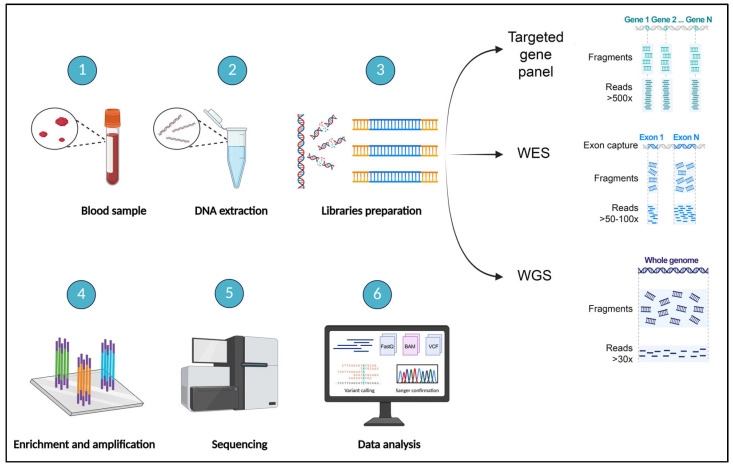
Overview of the NGS workflow and comparison of targeted gene panel, WES, and WGS approaches. (1) Blood sample collection: Peripheral blood is collected as a source of genomic DNA. (2) DNA extraction: Genomic DNA is isolated from the sample using standard extraction protocols. (3) Library preparation: DNA is fragmented, and sequencing adapters are ligated to create sequencing libraries. This step varies depending on the sequencing method used, as illustrated on the right side of the figure. (4) Target enrichment and amplification: Depending on the chosen method, regions of interest (ROIs) are captured using hybridization-based techniques. In targeted gene panels, a small set of disease-associated genes is enriched; in WES, all coding exons are captured; while WGS proceeds without enrichment. (5) Sequencing: The prepared libraries are sequenced using high-throughput platforms (e.g., Illumina), producing millions of short reads. (6) Data analysis: Bioinformatic analysis includes alignment of reads to a reference genome, generating Binary Alignment/Map (BAM) files and variant identification, typically reported in Variant Call Format (VCF). Downstream analyses include variant classification, confirmation (e.g., by Sanger sequencing), and clinical interpretation. This figure was generated using BioRender version June 2025 (https://BioRender.com). Created in BioRender. Treccarichi, S. (2025).

The implementation of NGS pipelines varies depending on the specific sequencing approach. In clinical diagnostics, it requires a well-structured workflow that integrates molecular biology procedures with bioinformatics analysis and clinical interpretation (Figure 2).

Each step in this process—from sample preparation to variant classification—plays a crucial role in ensuring the accuracy, reliability, and clinical relevance of the results. The technologies and tools adopted at each phase must be carefully selected and validated to maintain high diagnostic sensitivity and specificity. The key differences between targeted gene panels, WES, and WGS lie in the specific strategies used to capture the region of interest (ROI). Table 2 summarizes the main steps involved in the diagnostic workflows of these NGS approaches. These phases will be discussed in detail in the subsequent Section 2.1, Section 2.2, Section 2.3, Section 2.4, Section 2.5, Section 2.6, Section 2.7 and Section 2.8, with a focus on how they influence the performance and interpretation of targeted NGS panels in clinical settings.

### 2.2. Overview of NGS Workflows: Targeted Gene Panels, WES, and WGS

The workflow of NGS varies depending on the approach used—targeted gene panels, WES or WGS. While all methods share core steps such as DNA extraction, library preparation, sequencing, and data analysis, the main differences lie in the enrichment strategies and data interpretation. Targeted gene panels use hybridization or amplicon-based capture to isolate a predefined set of genes, allowing for high coverage and streamlined analysis. WES employs exome capture kits to enrich the coding regions of the genome, enabling broader variant detection across thousands of genes. In contrast, WGS requires no enrichment, providing an unbiased and comprehensive view of the genome, including intronic and intergenic regions. Each approach offers a unique balance of depth, breadth, and complexity, tailored to specific diagnostic needs.

### 2.3. Region of Interest (ROI) Capture in NGS

Region of Interest (ROI) capture is a defining feature that differentiates the main NGS approaches—targeted gene panels, WES and WGS. In targeted gene panels, the ROI is limited to a specific, clinically relevant set of genes, often selected based on phenotype or disease category, enabling high sequencing depth and efficient variant detection [31,32]. The selection of genes for inclusion in a diagnostic panel is a critical first step that directly affects both sensitivity and specificity. This selection is informed by curated gene–disease association databases (e.g., OMIM, GeneReviews, MalaCards), disease-specific consortia, and recent literature. In clinical diagnostics, phenotype-driven approaches are preferred: they allow for a streamlined panel that captures the most relevant genes, reducing the interpretation burden while maximising diagnostic yield. Genes are typically included based on evidence from clinical validity frameworks, frequency and nature of known pathogenic variants, inheritance mode, penetrance, and relevance to differential diagnosis [33,34]. In addition, initiatives such as ClinGen [35] provide robust guidelines and curated resources for selecting clinically relevant genes, dynamically updating panel content over time.

WES expands the ROI to include all protein-coding exons (about the 1–2% of the entire genome), using hybrid capture to target these regions across the genome, which is ideal for diseases with broader genetic heterogeneity [36]. Exome capture is typically performed using a hybridization-based method, where biotinylated DNA probes—designed to match known exonic sequences—are used to selectively bind coding regions within the DNA library. These probe-bound fragments are then isolated using streptavidin-coated magnetic beads, effectively enriching the sample for exonic content. After non-targeted sequences are washed away, the captured DNA is PCR-amplified and prepared for sequencing. Several commercial kits are widely used for this process, including Agilent SureSelect, Illumina Nextera Exome, Twist Human Core Exome, and Roche SeqCap EZ, each offering varying levels of coverage, target size, and capture efficiency.

In contrast, WGS does not involve selective capture; instead, it sequences the entire genome, covering both coding and non-coding regions [28]. This comprehensive ROI allows for the detection of structural variants, deep intronic mutations, and regulatory element disruptions that are often missed by panel or exome-based methods.

### 2.4. Library Preparation

Following DNA extraction, fragmentation is achieved through enzymatic digestion or sonication, aiming for a target insert size appropriate for the sequencing platform (typically 150–350 bp). The ligation of platform-specific adapters, often including unique molecular identifiers (UMIs), enables downstream amplification and indexing. The success of this step is contingent on high-molecular-weight DNA and accurate quantification. Quality control is performed using fluorometric assays (e.g., Qubit) and electrophoretic methods (e.g., Bioanalyzer or TapeStation) [37]. In-depth studies [38] describe the biases introduced during library preparation and offer strategies to mitigate them in clinical settings. As previously reported, a comparison was made between WES and WGS in terms of library preparation and exonic region coverage [39,40]. WES was performed using the Agilent SureSelect v5 + UTR capture kit, while WGS was conducted using a PCR-free library preparation. Both methods were applied to DNA samples from five female individuals. Sequencing was carried out on a HiSeq 2000 for WES (100× coverage) and a HiSeq X Ten for WGS (60× coverage). The results demonstrated that PCR-free WGS provides more uniform and complete coverage of exonic regions compared to WES, underscoring the benefits of PCR-free protocols in clinical sequencing applications.

As documented, library preparation for long-read sequencing methods—such as PacBio and Oxford Nanopore—differs significantly from short-read approaches. For example, PacBio requires a pre-library fragmentation step, often performed using the Megaruptor^®^ system (Diagenode), which shears high-molecular-weight DNA into controlled fragment sizes, typically ranging from 2 kb to over 100 kb [41]. Size selection is typically performed on the Sage BluePippin.

### 2.5. Target Enrichment

Target enrichment strategies vary depending on the NGS approach used in clinical genetics. For example, targeted gene panels isolate specific genomic regions of interest prior to sequencing, enhancing efficiency and achieving greater sequencing depth compared to non-targeted approaches. Hybridisation-based capture (e.g., Agilent SureSelect, Roche SeqCap) provides high specificity and is well suited for panels with >100 genes, while PCR-based methods (e.g., Ion AmpliSeq) offer faster turnaround and are ideal for smaller panels or FFPE (Formalin-Fixed, Paraffin-Embedded) samples. Hybridisation methods tolerate some degree of sequence divergence, aiding the capture of homologous genes or pseudogenes, though they require longer hands-on and hybridisation times [42]. Factors such as GC content, repetitive sequences, and probe design strongly influence enrichment efficiency. For WES, target enrichment is typically performed using the SureSelect Target Enrichment Reagent Kit [41].

### 2.6. Sequencing

Sequencing is performed using high-throughput NGS platforms capable of generating hundreds of millions of reads per run. Illumina platforms (e.g., NovaSeq, NextSeq) dominate clinical applications due to high accuracy, while Ion Torrent systems provide faster workflows at slightly reduced read quality. The required sequencing depth is typically ≥ 100× for germline diagnostics, though >500× may be necessary for mosaicism or low-frequency somatic variants. Base quality scores (Q-scores), duplication rates, and coverage uniformity are key metrics for evaluating run success [38,43]. For WES, the sequencing run can be performed on various platforms, including Illumina HiSeq 2500, HiSeq 3000/4000, and HiSeq 4000, due to their high throughput, accuracy, and compatibility with exome capture protocols [41].

### 2.7. Data Analysis

The bioinformatic processing of NGS data begins with raw FASTQ files, which contain the sequenced reads and their associated quality scores. These reads undergo quality control and trimming to remove low-quality bases and adapter sequences. The cleaned reads are then aligned to a reference genome (e.g., GRCh38) using alignment tools, generating SAM/BAM files. Post-processing steps, including duplicate marking, base quality recalibration, and indel realignment are performed to improve accuracy. Finally, variant calling is conducted using tools such as GATK, FreeBayes, or DeepVariant, producing a VCF (Variant Call Format) file that lists single nucleotide variants (SNVs), insertions, deletions, and other genomic alterations [44,45].

### 2.8. Variant Interpretation

**Figure 3:**
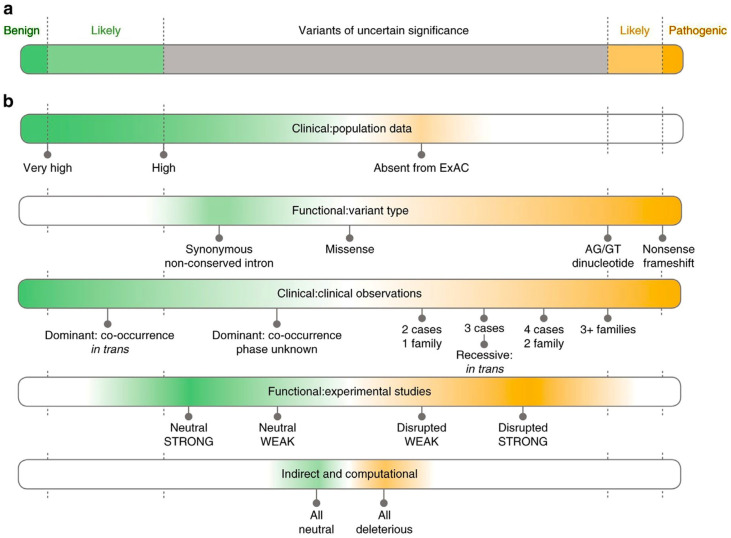
Schematic representation of variant classification based on ACMG criteria. (**a**) Flowchart illustrating the decision-making process used to classify genetic variants as pathogenic, likely pathogenic, variant of uncertain significance (VUS), likely benign, or benign, according to the ACMG guidelines. (**b**) Five categories of evidence are used in variant classification, including population data and clinical findings. Functional evidence encompasses sequence-level observations, molecular studies, and computational or indirect data. ExAC refers to the Exome Aggregation Consortium, a large population database commonly used in assessing variant frequency. Green represents benign variants, and orange pathogenic variants, with light green and light orange indicating likely benign and likely pathogenic, respectively. The white zone reflects intermediate or uncertain evidence, where available data do not strongly support either a benign or pathogenic classification. The label “All” refers to situations in which all computational tools provide concordant predictions (neutral or deleterious), supporting a confident classification as benign or pathogenic. This Figure was adapted from a previous work [46].

Interpreting the clinical significance of variants relies on multiple lines of evidence. Frequency data from gnomAD help exclude common benign variants, while ClinVar, HGMD (Human Gene Mutation Database), and LOVD (Leiden Open Variation Database) provide insight into clinical assertions. In silico tools (e.g., SIFT, PolyPhen-2, CADD) predict the functional impact of missense variants but should not be used in isolation. ACMG/AMP guidelines provide a structured scoring system for classification, incorporating segregation data, functional studies, conservation, and population frequency [19]. Conflicting classifications require expert consensus or multidisciplinary review. Figure 3 schematizes the different scoring thresholds used for variant classification in different categories.

Variant classification according to the ACMG guidelines is a standardized framework used to assess the clinical significance of genetic variants. This system categorizes variants into five classes: pathogenic, likely pathogenic, VUS, likely benign, and benign. Classification is based on multiple lines of evidence, grouped into categories such as population frequency data, computational predictions, functional studies, segregation analysis, and clinical phenotype correlation. Each piece of evidence is weighted as very strong, strong, moderate, or supporting, and a combination of criteria determines the final classification. This structured approach ensures consistency and reliability in the interpretation of genomic data in clinical diagnostics.

VUSs require particular caution in clinical genetics, as their interpretation is complex and depends on multiple factors. VUSs include diverse types with variable clinical implications. Missense variants are often difficult to interpret, while nonsense mutations may lead to premature stop codons and truncated proteins subject to nonsense-mediated decay [47]. In-frame insertions/deletions can alter splicing and gene function, and non-coding regulatory variants may affect gene expression, though their impact is still poorly understood. Only a minority of VUSs are ultimately reclassified as pathogenic, and their uncertainty complicates clinical decision-making, sometimes leading to unnecessary interventions and patient distress [48]. Efforts to improve interpretation and subclassification, along with family-based studies and enhanced counselling, can help mitigate these challenges. However, the balance of such strategies depends on the clinical context, highlighting the need for systematic and broadly supported policies.

Table 3 resumes the ACMG criteria used for the classification of the prioritized genetic variants.

The capacity to detect different classes of genetic variants differs substantially between targeted gene panels, WES, and WGS, reflecting their distinct design and scope. While single-nucleotide variants (SNVs) and small insertions/deletions (indels) are generally well detected across all three approaches, WGS achieves high sensitivity for SNV detection in coding regions when sequencing depth is adequate, as shown by Trudsø et al. [49], who reported comparable SNV detection performance between WGS, WES, and targeted panels in coding regions. Nonetheless, some low-coverage genomic regions may be less accurately detected than exonic regions covered by WES or targeted panels. By contrast, larger and more complex variants—including copy number variants (CNVs), structural variants (SVs), intronic/regulatory mutations, and repeat expansions—present specific challenges that differ across approaches. These distinctions are summarized in Table 4.

### 2.9. Clinical Correlation

Variant pathogenicity must be assessed in light of the patient’s clinical presentation. This includes detailed phenotyping, pedigree analysis, and segregation data. Discrepancies between genotype and phenotype may indicate reduced penetrance, variable expressivity, or dual diagnoses. Multidisciplinary team meetings (MDTs), involving clinical geneticists, molecular pathologists, and bioinformaticians, enhance diagnostic accuracy and facilitate re-evaluation of uncertain findings over time [50]. Parental testing can aid in phasing compound heterozygous variants or confirming de novo events. It should be stressed that international cooperation is of particular importance for the diagnosis of rare diseases, supporting interdisciplinary clinical discussions [51,52,53]. Clinical correlation is often supported by curated databases that compile genetic variants previously associated with specific phenotypes. Among the most widely used is the Human Gene Mutation Database (HGMD), which offers a comprehensive collection of published germline mutations linked to human inherited diseases [54]. ClinVar, maintained by the NCBI, is another essential resource that aggregates information about genomic variation and its relationship to human health [55]. It is community-curated, with users submitting variants along with their clinical classification, supporting evidence, and pathogenicity assessment criteria. Additionally, DECIPHER provides detailed phenotypic descriptions alongside potentially associated genetic variants, making it particularly useful for studying rare developmental disorders [56].

Gene-specific databases also support disease-focused interpretation. For instance, the SFARI Gene database categorizes genes associated with ASD [57], while PsyGeNET compiles information on genes and their associations with psychiatric disorders [58]. These resources are critical for guiding variant interpretation and supporting genotype–phenotype correlations in both research and clinical diagnostics.

### 2.10. Reporting and Counseling

The final clinical report includes only variants with established or probable clinical relevance and follows structured templates to ensure clarity and consistency. Reports highlight zygosity, predicted effect, classification (e.g., pathogenic, VUS), and evidence sources. They also state methodological limitations, such as inability to detect deep intronic variants or structural rearrangements. Genetic counselling is integral to discuss implications, psychosocial impact, reproductive options, and cascade testing for at-risk relatives. Counselling must be tailored to cultural context, literacy, and emotional state of the patient and family [4,59].

## 3. Clinical Applications of Targeted Gene Panels, WES and WGS

### 3.1. The NGS Techniques

#### 3.1.1. Diagnostic Application

Advancements in NGS techniques have transformed genetic diagnostics by enabling the detection of disease-causing variants with increasing breadth and resolution. Targeted gene panels, WES, and WGS each offer distinct advantages and are applied based on clinical context, diagnostic yield, and cost-effectiveness.

NGS panels represented a cornerstone in the diagnostic work-up of genetic diseases. Their clinical utility spans a wide spectrum of conditions, from well-defined monogenic syndromes to more heterogeneous and complex disorders. The following subsections highlight the main fields of application of NGS panels in clinical diagnostics, summarized in Table 5.

Currently NGS panels are highly effective in identifying mutations in genes responsible for single-gene disorders. Classical examples include cystic fibrosis (mutations in *CFTR* gene), Duchenne muscular dystrophy (*DMD* gene), and familial hypercholesterolemia (*LDLR*, *APOB*, *PCSK9* genes). These conditions, although genetically well-characterized, present with diverse clinical phenotypes and variable age of onset, making molecular confirmation essential for diagnosis, prognosis, and therapeutic decisions. NGS panels enable the simultaneous analysis of all coding exons and flanking intronic regions of disease-associated genes, significantly improving the diagnostic yield compared to traditional methods such as Sanger sequencing or Multiplex Ligation-dependent Probe Amplification (MLPA).

The high-throughput nature of NGS allows for rapid and parallel interrogation of multiple candidate genes, which is particularly advantageous in phenotypically overlapping syndromes or in cases with atypical manifestation. For instance, in cystic fibrosis, the use of NGS allows detection of both common and rare variants that may be missed by targeted mutation panels. Similarly, in Duchenne muscular dystrophy, comprehensive sequencing of the *DMD* gene can identify small insertions, deletions, and point mutations that are not detectable with MLPA alone. Combining MLPA and NGS has become the standard approach to achieve near-complete mutation detection in *DMD* [60,61,62,63]. By targeting the most commonly mutated loci, these panels increase diagnostic yield and reduce the time and cost associated with sequential testing. Ultimately, early and precise molecular diagnosis facilitates timely initiation of disease therapies and enables cascade testing, including familial risk assessment, reinforcing the clinical utility of NGS in the diagnostic workflow of monogenic disorders.

On the other hand, WES has become a widely adopted tool in clinical genetics for diagnosing rare and complex disorders [64]. WES is often performed as a trio analysis—sequencing the proband and both parents—to increase diagnostic accuracy and detect de novo variants. Its relatively high diagnostic yield, combined with decreasing costs and improved bioinformatics pipelines, has made WES a key component of routine clinical diagnostics. As documented, combining WES-Trio analysis with CNV detection significantly increases diagnostic yield [65,66,67]. While WES is primarily designed to identify SNVs and small indels within coding regions, it may miss larger deletions or duplications. Incorporating CNV analysis—using dedicated bioinformatic tools on WES data or complementary techniques like chromosomal microarray—allows for the detection of pathogenic structural alterations that contribute to a substantial proportion of genetic disorders, particularly in neurodevelopmental and syndromic conditions.

#### 3.1.2. NGS Applications: From Clinical Diagnosis to Cohort-Based Studies

It is important to distinguish between clinical diagnostic testing and cohort-level research analyses in genetics. At the individual level, genetic testing is performed with the primary goal of reaching a diagnosis that can directly guide patient management, prognosis, and counseling. Depending on the clinical presentation, this may involve targeted NGS panels, WES, or WGS. For instance, by selectively sequencing all protein-coding regions of the genome, WES enables the identification of pathogenic variants in both known and novel disease-associated genes. In fact, it is now widely used both in clinical diagnostics and in research settings. It is particularly valuable in cases with genetic heterogeneity, such as neurodevelopmental disorders (especially for ASD), intellectual disability, epilepsy, and congenital anomalies, where targeted gene panels may be insufficient [68,69].

For a gene to be used in genetic diagnosis, several criteria (supported by the ACMG guidelines) must be fulfilled to establish its validity as disease-causing. First, there must be a clear genotype–phenotype correlation, typically demonstrated by the segregation of the disease with the variant in affected families [70]. Second, the gene should harbour pathogenic or likely pathogenic variants with a strong functional impact, such as frameshift, nonsense, or canonical splice-site mutations, which are more likely to disrupt protein function. Third, the gene’s clinical relevance should be supported by its inclusion in specialized databases (e.g., OMIM, ClinVar, HGMD) that curate evidence linking genes to specific diseases [71]. Together, these criteria ensure that variants in the gene can be confidently interpreted in a diagnostic context.

**Figure 4:**
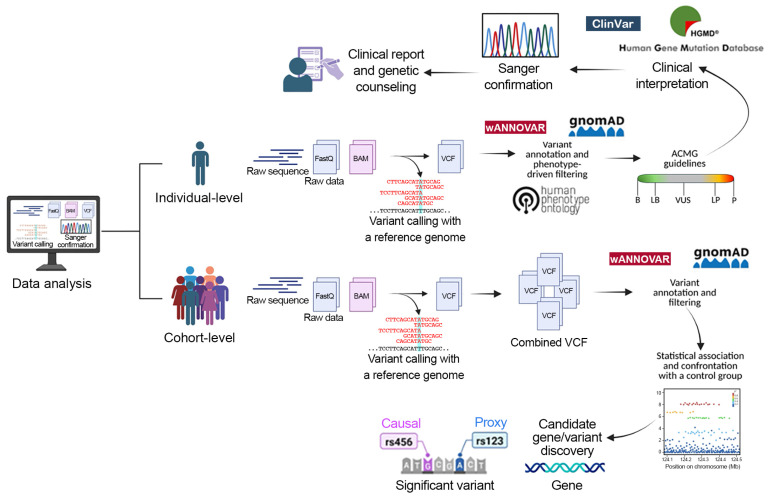
Data analysis workflows for sequencing studies at the individual and cohort levels. After sequencing, raw data (FASTQ files) are aligned to a reference genome to generate BAM files, followed by variant calling to produce VCF files. In individual-level analysis (clinical testing), variants undergo annotation and phenotype-driven filtering using resources such as gnomAD and the Human Phenotype Ontology (HPO). Variants are then classified according to ACMG guidelines and interpreted using databases such as ClinVar and HGMD. Pathogenic or likely pathogenic variants are confirmed by Sanger sequencing and reported in the clinical context with genetic counseling. In cohort-level analysis (discovery research), VCF files from multiple individuals are combined for joint analysis, annotated and filtered, and assessed for statistical association with disease traits. This approach enables candidate gene and variant discovery for further functional studies.

The outcome is the identification of a pathogenic or likely pathogenic variant that explains the patient’s phenotype, which is then included in the clinical report and used for medical decision-making. In contrast, cohort-level analyses are designed for research purposes, aiming to expand knowledge by identifying novel pathogenic variants or new disease-associated genes. Such studies typically involve sequencing data from groups of patients with similar but unresolved phenotypes, where VUS or rare, previously unreported mutations are investigated. In a genetic cohort study, genomic data (obtained through WES, WGS, or other approaches) are collected alongside clinical and environmental information. The goal is to determine whether, and in what way, genetic variants influence disease risk, progression, or treatment response. Beyond NGS, large-scale approaches such as genome-wide association studies (GWAS) and epigenome-wide association studies (EWAS) are also employed in research cohorts, enabling the discovery of susceptibility loci, regulatory elements, or epigenetic modifications that contribute to disease risk [72,73]. While these findings may not always provide an immediate diagnosis at the individual level, they represent valuable insights for refining diagnostic strategies, updating gene panels, and advancing precision medicine. Figure 4 graphically illustrates the workflows for sequencing studies at the individual and cohort levels.

### 3.2. Neurodevelopmental Disorders

According to the classification of the Diagnostic and Statistical Manual of Mental Disorders, Fifth Edition (DSM-5), neurodevelopmental disorders (NDDs) encompass a broad spectrum of heterogeneous conditions arising from abnormal brain development, often leading to impairments in cognition, communication, adaptive behavior, and psychomotor skills [74]. Currently, chromosomal microarray (CMA) is used as a first-tier diagnostic test to detect chromosomal abnormalities, and it is often complemented by *FMR1* CGG repeat expansion analysis to screen for Fragile X syndrome, one of the most common inherited causes of intellectual disability (ID).

#### 3.2.1. From Targeted Panels to NGS

Targeted gene panels were initially used as second-tier diagnostic test for NDDs. These panels include key genes such as *MECP2*, responsible for Rett syndrome, and *FMR1* [61,62,75,76]. They facilitated the identification of additional disease-associated loci, remaining clinically useful as knowledge expanded. For example, a 221 gene panel proved effective in establishing molecular diagnoses in 21% of cases, with higher yields observed in female patients and in specific phenotypes such as ASD and epilepsy [77]. In this study, 64.4% of cases showed de novo variants, and recurrently altered genes included *SLC2A1*, *SCN1A*, *ANKRD11*, *ATP1A2*, *CACNA1A*, *FOXP1*, and *GNAS*, together accounting for 19.7% of diagnoses.

Reported diagnostic yields of targeted NGS panels in neurodevelopmental conditions such as IDD, vary between 10% and 25%, depending on panel size, selection criteria and the patient cohort characteristics. For instance, the UK10K Project Rare Disease study reported an 11% yield using a 565-gene panel [60,63,78,79].

#### 3.2.2. Limitations of Targeted Panels

Although still clinically relevant, targeted NGS panels are now largely replaced by WES and WGS, which provide broader genomic coverage and higher diagnostic yield in genetically heterogeneous disorders. Compared to NGS panels, WES and WGS offer the advantage of identifying novel candidate genes potentially associated with NDDs, as well as detecting secondary mutations that may contribute to variable expressivity [80]. However, panels generally offer higher sequencing depth, useful for detecting low-level mosaicism. Their main limitation is the restricted gene content, often outdated as the number of NDD-associated genes grows rapidly [81]. For example, the SFARI Gene database currently lists over 1200 genes implicated in ASD (https://gene.sfari.org/) (accessed on 28 August 2025).

In clinical practice, WES is now the most widely used genetic test for diagnosing NDDs, and has even been proposed as a first-tier diagnostic tool, followed by array-CGH when necessary [82]. Another key difference between WES and targeted gene panels lies in their diagnostic yield across various clinical presentations of NDDs. As documented by a comprehensive meta-analysis, WES has demonstrated higher diagnostic yields in cases with dysmorphic features (54.7%), syndromic presentations (37.6%), and co-occurring epilepsy (35.6%). It also performs well in patients with early-onset epilepsy (32.3%), epileptic encephalopathy (34.7%), and drug-resistant seizures (25.4%), outperforming gene panels in these complex phenotypes [83].

Finally, WGS offers an even more comprehensive approach, detecting structural variants such as multi-exon deletions, contiguous gene deletions, and retrotransposon insertions, which typically escape WES. Despite the complexity of data interpretation, WGS achieves diagnostic yields exceeding 30% in NDDs [84]. Integrating CNV analysis into trio-based WES further increases yields: in one study of 187 patients, the diagnostic rate reached 40.11%, with nearly 7% of cases diagnosed solely through CNV [67]. This combined strategy proved cost-effective and clinically impactful, improving outcomes for ~40% of families.

#### 3.2.3. Novel Candidate Genes in NDDs

WES and WGS not only support clinical diagnosis but also contribute to the discovery of novel genes potentially involved in NDDs. This often occurs when VUS, or likely pathogenic/pathogenic variants, are detected in genes not previously—or only weakly—associated with the patient’s phenotype. While such findings are typically noted by the geneticist in the clinical report, they may not be directly linked to the individual’s condition. Instead, they represent valuable insights for research purposes, supporting gene discovery and expanding the understanding of NDD genetics. For instance, our recent findings highlight the involvement of *MAN2A2* in congenital disorder of glycosylation (CDG), defects related to abnormality in glycosylation, ASD and cognitive delay [85]. Another key finding from our WES studies is the association of two zinc finger genes, *ZC2HC1C* and *ZNF496*, with neurodevelopmental disorders (NDDs), particularly ASD [86,87]. We also identified the *PSMC5* gene [88] as being associated with neurodevelopmental phenotypes, further highlighting how the WES approach can improve diagnostic yield compared to targeted gene panels. Although *PSMC5* has been reported multiple times in the ClinVar and DECIPHER databases in association with specific neurodevelopmental features, it has not yet been linked to a defined MIM phenotype entry in the OMIM database. Additional genes identified through WES, such as *ARIH2* [89] and *FGFR2* [90], have also been proposed as potential contributors to ASD susceptibility.

It is worth noting that several NDDs such as ADHD are often influenced by environmental factors. For conditions like ADHD, a suitable genetic approach is the use of epigenome-wide association studies (EWAS), which allow for the identification of disease-associated DNA methylation profiles [91]. In addition, integrating EWAS findings with polygenic risk score (PRS) calculations can help better understand the complex interplay between genetic susceptibility and environmental influences in ADHD and related disorders. Despite the fact that several studies are needed, epigenetic variations are being widely studied as a molecular biomarker of complex NDDs and disease-related exposures.

Overall, NGS approaches play an indispensable role in genetic counseling, and enrollment in precision medicine trials tailored to NDDs. However, variant interpretation remains critical, particularly for variants of uncertain significance, whose pathogenicity often necessitates segregation studies, functional assays, or reanalysis over time [81]. Combined with clinical, neurophysiological, and imaging data, NGS techniques embody an integrated diagnostic paradigm essential for accurate genotypic–phenotypic correlation in NDDs.

### 3.3. Psychiatric Disorders

#### 3.3.1. Genetic Diagnosis by Using NGS Tools

Psychiatric disorders are complex mental health conditions that affect mood, cognition, behavior, and perception. They include disorders such as schizophrenia, bipolar disorder, and major depressive disorder. Psychiatric disorders often overlap with NDDs such as ASD and ADHD. These complex conditions share genetic overlap with traits such as brain structure, cognition, immunity, and cardiovascular disease, indicating a shared genetic etiology [92]. However, current polygenic risk scores lack clinical utility at the individual level. Given their high heritability and genetic complexity, next NGS approaches, particularly WES and WGS, have become valuable tools for identifying rare and common genetic variants associated with these conditions. Nevertheless, the widespread use of genetic testing for the clinical diagnosis of psychiatric disorders remains limited, largely due to the marked clinical and genetic heterogeneity of these conditions. By enabling the discovery of both monogenic forms and polygenic risk contributors, NGS continues to advance our understanding of the molecular basis of psychiatric disorders and supports the development of more personalized diagnostic and therapeutic strategies. The integration of NGS, epigenomic, and transcriptomic data through specialized databases related to psychiatric disorders represents a valuable resource for researchers [93]. These multi-omics platforms enable in-depth investigation of the pathophysiology and etiology of psychiatric conditions, supporting the identification of novel biomarkers and therapeutic targets. For this purpose, we can mention PD_NGSAtlas, PsyGeNET, PsychENCODE, and CommonMind Consortium [58,93,94,95].

The genetic diagnosis of psychiatric disorders is often complex. One of the most well-documented examples is the 22q11 deletion syndrome (DiGeorge syndrome), which is commonly detected using array-CGH, although WGS is increasingly applied for this purpose [96,97]. This recurrent deletion encompasses several genes, including *COMT* and *PRODH*. Variants in *COMT* affect catechol-O-methyltransferase activity (MIM #621296) and have been linked to psychiatric manifestations, while *PRODH* variants contribute to schizophrenia susceptibility (MIM #600850). Another important gene involving the 22q11.21 deletion is *TBX1*, which is associated with velocardiofacial syndrome (MIM #192430). This condition encompasses a broad spectrum of behavioral and psychiatric manifestations, including psychotic illness, paranoia, and aggression. Importantly, although identifying the 22q11 deletion is critical for the clinical diagnosis of DiGeorge syndrome, not all carriers develop psychiatric disorders, reflecting variable expressivity and incomplete penetrance [98]. According to the OMIM database, several genes have been linked to psychiatric disorders. However, in many cases—particularly for conditions such as schizophrenia—there is no strict one-to-one gene–disease relationship. Instead, many of these loci are described as susceptibility genes, meaning that genetic variants may increase an individual’s risk of developing the disorder but are not sufficient on their own to cause disease. This highlights the complex, multifactorial nature of psychiatric disorders, where genetic predisposition interacts with environmental and epigenetic factors.

#### 3.3.2. Novel Candidate Genes in Psychiatric Disorders

As widely documented, clinical translation in psychiatric genetics requires a multi-omics, cross-sectional approach that integrates genomic, transcriptomic, epigenomic, and proteomic data [99,100]. This comprehensive strategy allows for a deeper understanding of the complex biological mechanisms underlying psychiatric disorders and helps identify meaningful genotype–phenotype correlations. Within this context, as previously highlighted in multi-omic analyses, the *FURIN* and *SLC12A5* loci have been significantly associated with psychiatric outcomes [101].

Genome-wide association studies (GWASs) represent another valuable strategy for investigating genomic regions potentially linked to psychiatric disorders. For this purpose, several researchers have employed the PsychChip v1.0 array, which includes approximately 550,000 SNPs located in both exonic and intronic regions [102,103]. Based on GWAS analyses using this platform, several candidate genes have been identified, including *PDE1A*, *PPP1R1C*, *DAG1*, *QRICH1*, *RNF123*, *SMARCC1*, *IP6K2*, *IGSF11*, and *SORC3* [103]. Many of these genes are implicated in synapse development during fetal brain maturation, highlighting their potential relevance in the pathogenesis of psychiatric conditions.

WES is a valuable tool for identifying susceptibility genes potentially linked to psychiatric disorders. In our study, a key finding was the association of *CSMD1* as a potential contributor to psychiatric phenotypes, particularly in individuals presenting with borderline intellectual functioning (BIF) and obsessive–compulsive traits [43,104]. Additional genes identified in association with psychiatric disorders included *UNC5C*, *KLF13*, *PPP2R5E*, and *FAAH2*, further supporting the utility of WES in uncovering novel genetic contributors to complex neuropsychiatric conditions [105,106,107,108].

### 3.4. Neuromuscular Disorders

NGS is widely applied in the clinical diagnosis of neuromuscular disorders affecting both central and peripheral nervous system [109]. In particular, tailored gene panels have significantly improved diagnostic accuracy for these inherited conditions by enabling targeted analysis of relevant genes. Targeted NGS gene panels are commonly used for clinical diagnosis, as they enable rapid analysis of the most relevant genes, such as *DMD* (Duchenne/Becker muscular dystrophy), *SMN1* (spinal muscular atrophy), *RYR1* and *CACNA1S* (congenital myopathies and malignant hyperthermia susceptibility), *TTN* (titinopathies), *COL6A1*-3 (collagen VI-related myopathies), and *GNE* (hereditary inclusion body myopathy). Panels can achieve diagnostic yields of 30–50% depending on the phenotype [110]. They are widely adopted as first-line diagnostics for disorders such as Charcot–Marie–Tooth disease (CMT), spinal muscular atrophy (SMA), and early-onset forms of Huntington’s disease, offering faster and more cost-effective alternatives to WES or WGS approaches [111,112]. Pre-curated gene panels can be easily obtained from Invitae (San Francisco, CA, USA), including a comprehensive neuromuscular disorders panel (covering up to 230 genes) for patients with suspected skeletal muscle or neuromuscular junction disorders, and a comprehensive neuropathies panel (covering up to 111 genes) for those with suspected peripheral nerve or motor neuron disorders. Additionally, they represent a valuable tool for the clinical diagnosis of early-onset neuromuscular disorders [113]. In CMT, panel testing has achieved molecular diagnoses in approximately 6–46% of cases, uncovering both small sequence variants and copy number alterations not detected by standard techniques like MLPA or Sanger sequencing [114,115]. Similarly, a Polish cohort with varied neuromuscular phenotypes demonstrated a diagnostic yield of 55.8% using an 89-gene panel combined with MLPA, highlighting the relevance of including both sequence and structural variant detection [116].

*DNAJB6* is another important gene in the clinical diagnosis of muscular dystrophy (MIM #603511), although its underlying pathogenic mechanism remains incompletely understood [117]. NGS approaches are frequently employed in the clinical diagnosis of congenital titinopathies, a term recently proposed to describe a spectrum of *TTN*-related disorders [118] (known as titinopathies), including early-onset myopathy with fatal cardiomyopathy, centronuclear myopathy with cardiac involvement, and arthrogryposis multiplex congenita with myopathy. Pathogenic variants in the *TTN* gene can be detected using any of the main NGS approaches—targeted gene panels, WES, or WGS. A similar example involves *RYR1* gene variants, which are also identifiable through all NGS strategies, either as primary findings in patients with congenital myopathies or as incidental findings in unrelated clinical investigations [119]. For patients in whom panel testing is inconclusive, WES and WGS provide broader coverage and allow the identification of novel candidate genes. Importantly, genetic confirmation not only supports diagnosis and prognosis but also guides genetic counseling and eligibility for gene-targeted therapies.

### 3.5. Connective Tissue Disorders

Genetic testing for connective tissue disorders increasingly relies on NGS multi-gene panels. These panels are designed to analyze simultaneously a wide range of genes implicated in syndromes with overlapping clinical features, such as Marfan syndrome (MFS), Loeys–Dietz syndrome, vascular Ehlers–Danlos syndrome, and other heritable thoracic aortic diseases. Typically, they include genes such as *FBN1*, *TGFBR1*, *TGFBR2*, *SMAD3*, *TGFB2*, *COL3A1*, and *ACTA2*, among others, depending on the clinical suspicion. Compared with single-gene testing, panel-based approaches offer greater efficiency, reduced costs, and an improved diagnostic yield, especially in patients with atypical presentations. However, they may also generate variants of uncertain significance, requiring careful interpretation by clinical geneticists. In practice, targeted panels or broader connective tissue disorder panels are selected according to the patient’s phenotype, ensuring a personalized diagnostic strategy.

#### 3.5.1. Marfan Syndrome and Related Disorders

Marfan syndrome (MFS) is a multisystemic autosomal dominant connective tissue disorder primarily affecting the skeletal, cardiovascular, and ocular systems. Genetic testing plays a pivotal role not only in confirming the clinical diagnosis but also in risk stratification, family counselling, and guiding lifelong surveillance protocols [120]. Early molecular diagnosis is especially important to prevent catastrophic events such as aortic dissection and rupture, which remain the leading causes of mortality in MFS patients [121].

Targeted NGS gene panels have emerged as a powerful and time-efficient tool to detect pathogenic variants associated with MFS and related aortopathies. Compared to traditional Sanger sequencing, these panels provide a faster, cost-effective alternative that allows simultaneous analysis of multiple genes with high coverage and sensitivity [122]. Furthermore, NGS facilitates the identification of novel or atypical mutations and supports genotype–phenotype correlations that may inform prognosis and treatment. The *FBN1* gene, encoding fibrillin-1, accounts for the majority of classical Marfan syndrome cases, with mutations identified in approximately 80–90% of patients [120,123]. Fibrillin-1 is a glycoprotein crucial for the formation of extracellular microfibrils that provide structural support and regulate transforming growth factor-beta (TGF-β) bioavailability. Loss-of-function and dominant-negative mutations in *FBN1* disrupt microfibrillar integrity and dysregulate TGF-β signalling, contributing to the pathogenesis of aneurysms and skeletal abnormalities [124,125,126]. *FBN1* variants include a broad spectrum of mutations—missense, nonsense, splice-site mutations, small insertions/deletions, and large rearrangements—making comprehensive sequencing essential. NGS panels typically cover all exons and canonical splice sites of *FBN1*, often integrating algorithms for detecting structural variants and copy number changes [127,128]. The identification of certain “hotspot” mutations or variant clusters in domains critical for calcium-binding and protein folding (e.g., cbEGF domains) may also help predict clinical severity [129].

#### 3.5.2. Differential Diagnoses and Additional Genes

Marfan syndrome shares overlapping phenotypic features with other connective tissue disorders, notably Loeys–Dietz syndrome (LDS), vascular Ehlers–Danlos syndrome (vEDS), and familial thoracic aortic aneurysm syndromes. A precise molecular diagnosis is therefore critical to distinguish among these entities, each of which may have distinct prognostic implications and management strategies [130]. To facilitate differential diagnosis, most diagnostic NGS panels for thoracic aortic disease include a curated selection of additional genes, such as

- *TGFBR1* and *TGFBR2*: encoding receptors for TGF-β, are commonly mutated in LDS types 1 and 2. LDS is characterised by aggressive arterial aneurysms, hypertelorism, and bifid uvula or cleft palate. Mutations often exert dominant-negative effects on TGF-β signalling [131].
- *SMAD3*: an intracellular transducer of TGF-β signalling, is implicated in Aneurysm–Osteoarthritis Syndrome (AOS). Mutations in *SMAD3* can result in early-onset osteoarthritis, arterial tortuosity, and aneurysms [132].
- *COL3A1*: encoding type III procollagen, is the causative gene in vEDS. This condition poses high risk for spontaneous arterial and organ rupture, often in young adulthood. *COL3A1* variants are typically missense substitutions affecting glycine residues in the collagen triple helix domain [133,134].
- *ACTA2*: encoding smooth muscle α-actin, is frequently mutated in familial thoracic aortic aneurysms and dissections (FTAAD). Variants impair contractile function of vascular smooth muscle cells, predisposing to early aortic events and other cerebrovascular complications [135].

Comprehensive gene panels encompassing these genes are critical not only for accurate diagnosis but also for risk assessment in asymptomatic carriers. Furthermore, expanded panels may include other emerging candidates such as *MYH11*, *MYLK*, and *PRKG1*, which have been associated with heritable aortopathies and variable phenotypic expression [136,137].

### 3.6. Cardiovascular Disorders and Cardiomyopathies

Inherited cardiomyopathies constitute a heterogeneous group of cardiovascular disorders with a genetic basis, characterized by variable clinical phenotypes and frequently associated with an increased risk of malignant ventricular arrhythmias and sudden cardiac death (SCD). Among the most well-characterized conditions in this group are hypertrophic cardiomyopathy (HCM), long QT syndrome (LQTS), and Brugada syndrome (BrS). These disorders are primarily caused by rare, pathogenic variants in genes encoding sarcomeric proteins in hypertrophic cardiomyopathy, or in genes coding for ion channel subunits and their regulatory proteins in LQTS and Brugada syndrome, with autosomal dominant inheritance being the most frequently observed mode of transmission [138]. The implementation of targeted NGS panels have significantly improved the diagnostic yield in patients with suspected inherited cardiac conditions enabling the simultaneous analysis of multiple genes known to be associated with these phenotypes. The use of such panels also shortens the time to diagnosis and supports comprehensive genotype–phenotype correlation, which is essential for accurate risk stratification and clinical management [139,140]. Recent evidence also highlights the value of advanced sequencing approaches in refining the diagnostic landscape of inherited cardiomyopathies. In particular, a comprehensive genomic analysis recently reported by Zhang et al. [141] further supports the integration of broad NGS strategies to capture the genetic heterogeneity of these disorders. Table 6 summarizes key genes commonly associated with cardiomyopathies and frequently included in diagnostic NGS panels, along with relevant genomic information.

#### 3.6.1. Gene-Specific and Clinical Correlates

HCM is the most common monogenic cardiovascular disease, with an estimated prevalence of 1:500, though recent genome-first studies suggest higher subclinical rates [142,143]. In the case of HCM, pathogenic variants are found in genes encoding sarcomeric proteins such as *MYH7* and *MYBPC3*, which together account for approximately 50–60% of genotyped cases. The identification of these variants has prognostic implications, as certain genotypes have been linked to more severe phenotypes and earlier disease onset. Additional genes such as *TNNT2*, *TNNI3*, *TPM1*, and *ACTC1* contribute to the broad phenotypic and prognostic heterogeneity of HCM [144].

Similarly, in LQTS, variants in genes such as *KCNQ1*, *KCNH2*, and *SCN5A* are associated with distinct electrocardiographic patterns, triggers for arrhythmias, and differential response to β-blocker therapy. Brugada syndrome is most often associated with loss-of-function variants in *SCN5A*, although its genetic basis remains elusive in a significant proportion of patients [139].

Arrhythmogenic Cardiomyopathy (ACM) is a genetically determined myocardial disease characterized by fibrofatty myocardial replacement, ventricular arrhythmias, and progressive ventricular dysfunction. Traditionally known as arrhythmogenic right ventricular cardiomyopathy (ARVC), the classification has evolved to ACM to reflect its broader phenotypic presentations, including left-dominant and biventricular forms [145]. It is often caused by mutations in genes encoding desmosomal proteins such as *PKP2*, *DSG2*, *DSC2*, *JUP*, and *TMEM43*, especially in the classical right-dominant form [146].

#### 3.6.2. Diagnostic Yield and Clinical Utility of NGS

Beyond gene-specific findings, NGS approaches have been systematically evaluated for their diagnostic yield and clinical impact in inherited cardiomyopathies. Recent studies have shown that WES achieves a diagnostic yield exceeding 26% for inherited cardiovascular diseases, compared with approximately 18% using commercially available targeted gene panels [147]. In the context of periventricular heterotopia (PVH), several genes have been identified as contributing factors. Notably, mutations in the *FLNA* gene (OMIM #300017), as well as in *COL5A1*, *COL5A2*, and *COL3A1*, have been reported [147,148]. These collagen-related genes are primarily associated with Ehlers–Danlos syndrome (EDS), highlighting the clinical and genetic overlap between connective tissue disorders and cardiomyopathies. On the other hand, the *TNNI3* gene has been associated with cardiomyopathy, particularly with hypertrophic and restrictive subtypes [147]. It is worth mentioning that *TNNI3* gene is included in the gene panels for cardiomyopathy which are commonly used in pediatric diagnostics to identify genetic causes of early-onset cardiac conditions [149,150]. As previously indicated in Section 3.2, *TTN* and *RYR1* are additional key genes frequently implicated in neuromuscular and cardiomyopathic phenotypes. *TTN* variants are known to cause a broad spectrum of titinopathies, affecting both skeletal and cardiac muscle, while *RYR1* mutations are primarily linked to congenital myopathies and may also present with variable cardiac involvement.

The clinical utility of genetic testing in inherited cardiomyopathies is well established. It not only confirms the diagnosis in individuals with borderline or atypical clinical features but also facilitates the implementation of life-saving interventions, such as implantable cardioverter-defibrillators (ICDs) in high-risk patients, and lifestyle modifications or pharmacological therapy in those with modifiable risk factors. Furthermore, genetic testing enables the identification of asymptomatic mutation carriers within families, who may benefit from individualized surveillance protocols and preventive strategies, ultimately reducing mortality associated with these inherited disorders.

#### 3.6.3. Commercially Available Panels for Cardiomyopathies

Several commercial NGS panels are available for the molecular diagnosis of cardiomyopathies, offering varying gene content, coverage, and bioinformatic support. These panels typically include core sarcomeric genes (*MYH7*, *MYBPC3*, *TNNT2*, *TNNI3*, *TPM1*, etc.) and extend to non-sarcomeric and syndromic genes, depending on clinical indications. Examples include

- Invitae Cardiomyopathy Comprehensive Panel, which includes over 100 genes covering HCM, DCM, ACM, RCM, and LVNC. Key genes include *MYH7*, *MYBPC3*, *TNNT2*, *LMNA*, *TTN*, *PKP2*.
- Blueprint Genetics Cardiomyopathy Panel, offering high-coverage sequencing for ~150 genes with clinical-grade interpretation. Broad set includes sarcomeric, desmosomal, metabolic genes.
- Fulgent Cardiomyopathy Panel, focused on both adult and paediatric cardiomyopathies, including metabolic and mitochondrial genes. Key genes include *MYBPC3*, *MYH7*, *TNNI3*, *SCN5A*, *BAG3*.
- Centogene Cardio Genetics Panels, tailored for comprehensive or phenotype-specific testing with support for rare disease interpretation. Panel is customisable, and includes rare disease genes.
- CeGaT Cardiomyopathy Panel, designed with sub-panels for precise differential diagnosis, e.g., “Sarcomeric,” “Desmosomal,” or “Metabolic” subsets.

These panels differ in terms of technical parameters (e.g., minimum coverage, detection of CNVs or intronic variants), and selection should be guided by clinical phenotype, family history, and availability of post-test counselling [151]. Laboratories also offer trio-based testing or reanalysis options, particularly useful in unresolved or pediatric cases.

Overall, commercial NGS panels streamline the diagnostic pathway, supporting timely and cost-effective genetic diagnosis in routine clinical cardiology [152].

### 3.7. Inherited Cancer Syndromes

Comprehensive gene panels have become a cornerstone in the evaluation of hereditary cancer susceptibility, particularly in individuals with a significant family history of malignancy. These panels assess variants across a spectrum of genes associated with well-established cancer predisposition syndromes. Among the most frequently analysed are *BRCA1* and *BRCA2*, which are linked to hereditary breast and ovarian cancer syndrome, as well as mismatch repair genes such as *MLH1*, *MSH2*, *MSH6*, and *PMS2*, which are implicated in Lynch syndrome. Numerous studies have demonstrated that the inclusion of both high- and moderate-penetrance genes in multigene panels significantly enhances diagnostic sensitivity and the effectiveness of genetic counselling [153,154,155]. The integration of these genes enables the detection of a broader spectrum of pathogenic variants, which might otherwise be missed if only high-penetrance genes were assessed. This comprehensive approach facilitates a more accurate risk stratification for patients and their families, allowing for tailored surveillance protocols, risk-reduction strategies, and therapeutic decision-making.

Furthermore, ongoing advancements in NGS technologies have substantially expanded the breadth and depth of genetic testing. These technological improvements have led to the inclusion of an increasing number of genes associated with hereditary cancer syndromes, including those with lower penetrance or more recently identified roles in cancer predisposition. The enhanced analytical capacity allows for the identification of pathogenic variants in genes that were previously underrepresented or excluded from routine testing panels [156].

### 3.8. Metabolic Disorders

Metabolic disorders are a group of inherited conditions caused by defects in the biochemical pathways responsible for processing nutrients, producing energy, or eliminating waste. These disorders often result from pathogenic variants in genes encoding enzymes, transporters, or cofactors involved in metabolism. During the last decades, NGS—particularly targeted panels and WES—has significantly improved the diagnostic yield in metabolic disorders by enabling the rapid identification of causative variants across multiple genes. Early and accurate diagnosis is crucial, as many metabolic disorders are treatable, and timely intervention can prevent severe complications or irreversible damage.

As documented in a previous study utilizing a well-designed NGS panel in 48 subjects, several putative genetic variants were identified across multiple genes associated with common metabolic disorders [157]. Variants in *COL6A2*, *FTO*, *SPARC*, and *MTHFR* were linked to central obesity. Additionally, 17 patients (35.4%) carried variants in *APOB*, *SLC2A2*, *LPA*, *ABCG5*, *ABCG8*, and *GCKR*, which are associated with hyperglycemia. Three patients (6.3%) had variants in *APOA1*, *APOC2*, *APOA4*, and *LMF1*, related to hypertriglyceridemia. Eight patients (16.7%) showed variants in *ABCA1*, *CETP*, *SCARB1*, and *LDLR*, linked to low HDL-cholesterolemia, and five patients (10.4%) had variants in *ADD1*, associated with hypertension. This NGS panel was designed for diagnostic purposes targeting known disease-associated genes, rather than for cohort-level discovery of novel variants.

#### 3.8.1. Inborn Errors of Metabolism (IEMs)

Across the wide spectrum of metabolic disorders, NGS has significantly enhanced the diagnostic accuracy and efficiency for identifying inborn errors of metabolism (IEMs), including phenylketonuria (PKU), Gaucher disease, and maple syrup urine disease (MSUD). These rare genetic disorders are caused by pathogenic variants in genes encoding metabolic enzymes, leading to the accumulation of toxic metabolites or deficiencies in essential biochemical pathways [158].

NGS enables simultaneous analysis of multiple genes associated with metabolic disorders, which is particularly beneficial given the phenotypic overlap and genetic heterogeneity common among IEMs [159]. For example, phenylketonuria, one of the most common amino acid metabolism disorders, results from mutations in the *PAH* gene encoding phenylalanine hydroxylase. Early detection through molecular analysis allows prompt dietary intervention, preventing severe neurological damage [160].

Similarly, Gaucher disease, a lysosomal storage disorder caused by mutations in the *GBA* gene, can be efficiently diagnosed using NGS panels that include lysosomal and sphingolipid metabolism-related genes. Molecular diagnosis enables timely initiation of enzyme replacement therapy, which can significantly alter disease progression and improve patient outcomes [161].

In the case of maple syrup urine disease, a disorder of branched-chain amino acid catabolism due to defects in the *BCKDHA*, *BCKDHB*, or *DBT* genes, NGS facilitates rapid identification of the underlying mutation, which is crucial for early dietary management and prevention of metabolic crises [162]. Overall, the integration of NGS into clinical diagnostics has transformed the landscape of IEM detection, allowing for earlier diagnosis, tailored therapeutic strategies, and improved prognostic counselling for affected families.

#### 3.8.2. Mitochondrial Disorders

Mitochondrial disorders arise from defects in the function of mitochondria, the organelles responsible for producing energy in the form of ATP through oxidative phosphorylation. Because mitochondria play a central role in cellular energy metabolism, dysfunction in these pathways directly impacts various metabolic processes. These disorders are genetically, and clinically heterogeneous conditions caused by mutations in either the mitochondrial DNA (mtDNA) or in nuclear genes encoding mitochondrial proteins. While WGS remains the gold standard for detecting mtDNA variants, targeted NGS panels including nuclear-encoded mitochondrial genes (e.g., *POLG*, *SURF1*) are particularly valuable when direct mtDNA testing is limited by phenotype complexity or tissue availability [163]. In infants or critically ill patients, invasive procedures such as muscle biopsy may be unfeasible, making blood-based targeted panels a practical and less invasive and more first-tier diagnostic option. A significant proportion of mitochondrial diseases are caused by pathogenic variants in nuclear genes. These genes encode structural and regulatory components essential for mitochondrial function and effectively analyzed through targeted NGS. Current panels encompass over 200–300 nuclear genes involved in mitochondrial biogenesis, metabolism, and maintenance [164]. In a large cohort study of 450 patients, targeted NGS achieved a diagnostic yield of 30%, with 82% of pathogenic variants located in nuclear genes accounting for about two-thirds of all resolved cases (20%), while mtDNA variants explained the remaining 10%. These findings highlight the central role of nuclear genome analysis in the diagnosis of mitochondrial disorders and support the integration of nuclear gene panels into routine diagnostic workflows [165].

Recent evidence also supports dual-genome approaches that simultaneously assess both nuclear and mitochondrial DNA. These have proven superior to sequential or isolated testing. For example, a large-scale study, which applied a combined NGS panel to over 1500 patients with suspected mitochondrial disease, combined NGS panels achieved a 14.6% diagnostic yield, with variants evenly split between the two genomes [166]. Similarly, parallel analysis of WES and mtDNA in more than 2200 patients, resulting in improved diagnostic yield compared to WES alone, including Single Nucleotide Variants (SNVs) and CNVs, as well as mtDNA mutations [167]. These results support integrated testing as a first-tier strategy in suspected mitochondrial disorders.

Nonetheless, targeted panels have limitations. They may fail to detect low-level heteroplasmic mtDNA variants (below 10–15% variant allele frequency) and large mtDNA rearrangements, such as deletions or duplications. In such cases, complementary techniques like long-range PCR, Southern blot, or MLPA remain essential for detecting structural mtDNA variants [168].

In summary, targeted NGS panels represent an effective, less invasive, and cost-efficient diagnostic tool for mitochondrial disorders, particularly those involving nuclear gene mutations. When integrated with complementary or dual-genome approaches, they offer substantial clinical value in guiding diagnosis, management, and genetic counseling.

#### 3.8.3. Novel Candidate Genes in Metabolic Disorders

GWAS and, more recently, EWAS have been extensively applied to metabolic disorders such as obesity, type 2 diabetes, dyslipidemia, and hyperglycemia. GWAS has identified hundreds of common variants in loci including *FTO*, *TCF7L2*, *MC4R*, and *SLC30A8*, which influence traits such as body mass index, insulin secretion, and lipid metabolism [169,170,171]. For example, the *FTO* gene has been linked to obesity susceptibility (MIM #612460), *TCF7L2* to type 2 diabetes susceptibility (MIM #125853), *MC4R* to resistance to obesity (MIM #618406), and *SLC30A8* to non-insulin-dependent diabetes mellitus susceptibility (MIM #125853). These associations underscore the polygenic and multifactorial nature of metabolic diseases, where numerous genetic risk factors interact with environmental influences. Importantly, such findings generally reflect susceptibility loci rather than direct gene–disease causation, meaning that single variants cannot be used to establish a specific molecular diagnosis.

EWAS adds another layer of insight by uncovering disease-associated DNA methylation patterns and gene–environment interactions, providing potential biomarkers for metabolic risk and progression. While genome-wide approaches are not yet routinely used in clinical diagnostics, they represent a powerful tool for unraveling the complex genetic architecture of metabolic disorders and for identifying novel therapeutic targets.

As documented in other studies, WES analysis was very helpful for the identification of potential susceptibility genes for obesity, such as *LAMB3*, *LEPR*, and *UCP2* [172,173,174]. As outlined in another WES-based study, the *FAAH* and *DGAT2* genes—both involved in fatty acid biosynthesis—have been implicated in the development of human obesity [175]. Furthermore, the WGS approach has proven useful in identifying genetic variants associated with obesity. Notably, variants in the *CYB5A* and *CCND2* genes have shown significant associations with obesity, highlighting the value of WGS in uncovering novel genetic contributors to complex metabolic traits [176,177].

Overall, these genome-wide approaches provide valuable insights into genetic susceptibility and disease mechanisms but remain primarily research tools, not diagnostic methods for individual patients.

### 3.9. Neurodegenerative Disorders

#### 3.9.1. Clinical Applications of NGS

Neurodegenerative disorders are a group of progressive, debilitating disorders characterized by the gradual loss of structure or function of neurons, commonly affecting memory, movement, or cognition [178]. Conditions such as Alzheimer’s disease, Parkinson’s disease, amyotrophic lateral sclerosis (ALS), mild cognitive impairment (MCI) and Lewy body dementia (LBD) can be caused by both environmental and genetic factors. Neurodegenerative diseases can be classified according to primary clinical features (e.g., dementia, parkinsonism, or motor neuron disease), anatomic distribution of neurodegeneration (e.g., frontotemporal degenerations, extrapyramidal disorders, or spinocerebellar degenerations), or principal molecular abnormality [179,180]. Although these conditions often share overlapping features, they are heterogeneous in their clinical presentations and underlying pathophysiology. Each disorder is characterized by distinct epidemiological patterns, clinical symptoms, laboratory and neuroimaging findings, neuropathological changes, and management strategies [180].

NGS approaches—particularly gene panels, WES, and WGS—have greatly advanced the identification of causative mutations and genetic risk factors, facilitating earlier diagnosis, better understanding of disease mechanisms, and the potential for personalized therapeutic strategies.

Gene panels were among the first approaches used for the genetic diagnosis of neurodegenerative disorders. As previously documented, the MRC Dementia Gene Panel was developed to assess 16 genes known to harbor pathogenic mutations associated with dementia [181]. This panel included key genes such as *PRNP*, *PSEN1*, *PSEN2*, *APP*, and *MAPT*. Table 7 lists these genes and further insights related to the MRC Dementia Gene Panel.

While the panel was rapid, accurate, and cost-effective, its utility was limited by the small number of genes analyzed. With the continued discovery of novel gene–disease associations, the genetic landscape of neurodegenerative disorders has expanded significantly. According to the MalaCards database (https://www.malacards.org/) (accessed on 28 August 2025), 762 genes are currently associated with dementia, with 67 genes having an association score above 100—highlighting the growing genetic complexity of these conditions.

A more recent targeted gene panel including 90 dementia-related genes was developed in 2021 and applied in a Taiwanese cohort of patients [182]. This panel, combined with plasma amyloid biomarker analysis, demonstrated a high diagnostic yield. Among 52 patients with young-onset AD, 9 patients (17.3%) carried pathogenic mutations: 2 in *APP*, 4 in *PSEN1*, 2 in *PSEN2*, and 1 in *TREM2*. In addition, 2 of 33 patients (6.1%) with young-onset frontotemporal dementia (FTD) had mutations in *MAPT* and *LRRK2*, while 3 of 6 patients (50%) with possible FTD combined with other neurodegenerative disorders had mutations in *APP*, *PSEN2*, or *MAPT*. Notably, individuals with *PSEN1* mutations exhibited an earlier disease onset compared to those without mutations.

Although gene panels are still widely used for the genetic diagnosis of neurodegenerative disorders, WES offers important advantages [183,184]. Unlike panels, which focus only on a predefined set of clinically relevant or research-driven genes, WES enables the analysis of nearly all protein-coding genes, thereby expanding the potential to uncover novel variants of interest.

#### 3.9.2. Novel Candidate Genes in Neurodegenerative Disorders

In a research setting, WES can be particularly effective, provided that sufficient sequencing depth is achieved in the genes of interest. Within this context, results from a WES study identified several new genes significantly associated with neurodegenerative disorders, including *SORL1*, *GRID2IP*, *WDR76*, and *GRN* [185]. *SORL1* has been linked to amyloid precursor protein processing and Alzheimer’s disease risk, while *GRN* mutations are a well-established cause of frontotemporal dementia. *GRID2IP* and *WDR76* are more recent candidates, with emerging evidence suggesting roles in synaptic function and neuronal survival. In another study, WES facilitated the identification of novel variants in genes potentially associated with neurodegenerative conditions, including *SQSTM1*, *NOS3*, *ABCA7*, *ERBB4*, *SETX*, *NEFH*, and *ADCM10* [186]. These findings highlight how WES can uncover both known and novel contributors to the genetic architecture of neurodegenerative diseases.

Concerning WGS, it can be applied as a single comprehensive genetic test to detect a wide range of clinically relevant variants in patients with dementia phenotypes. In one study, it was successfully used for the genetic diagnosis of early-onset dementia, demonstrating that WGS as a first-line diagnostic tool can both increase diagnostic yield and shorten time to diagnosis [187]. A single WGS analysis enables accurate detection of *C9orf72* repeat expansions, as well as the identification of single nucleotide variants and expanded short tandem repeats (STRs), underscoring its clinical utility in neurodegenerative disorders.

### 3.10. Clinical Application and Diagnostic Choice

The selection of the most appropriate genomic test is driven by the clinical context, disease characteristics, and the balance between diagnostic yield, turnaround time, and cost. In well-characterized monogenic disorders with a defined set of causative genes, targeted multigene panels remain the most efficient first-line approach. They provide rapid and cost-effective results, directly informing diagnosis and patient management.

**Figure 5:**
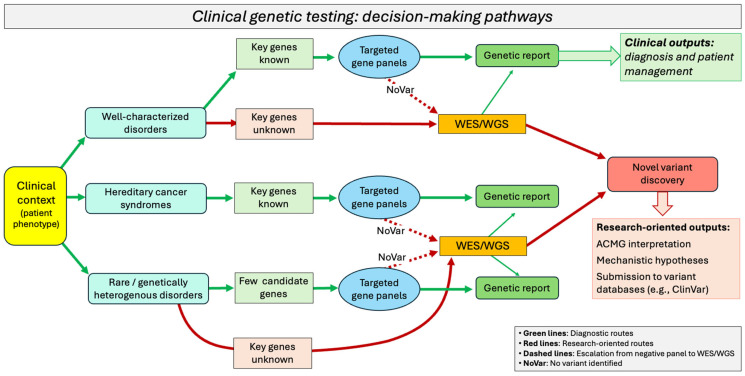
Clinical genetic testing: decision-making pathways. Clinical context determines whether targeted gene panels, WES, or WGS are applied. Panels are preferred for well-characterized disorders and hereditary cancer syndromes, while WES/WGS allow discovery of rare or heterogeneous conditions. Clinical outputs include genetic reports guiding diagnosis and patient management; research-oriented outputs include novel variant discovery, variant interpretation (ACMG), mechanistic hypotheses, and submission to databases (e.g., ClinVar). Dashed lines indicate alternative pathways, applied when targeted panels fail to identify causal variants, prompting escalation to WES and/or WGS. In practice, WES and WGS may function either sequentially or as alternative first-line approaches, depending on clinical context and availability. Importantly, while WES/WGS enable novel variant discovery and research-oriented outputs, they also provide clinically actionable reports for diagnosis and patient management.

When targeted testing does not identify a causal variant or when the key genes underlying the phenotype are unknown, escalation to whole-exome sequencing (WES) offers a broader, hypothesis-free exploration of coding regions. WES is particularly valuable in rare or genetically heterogeneous conditions, where multiple candidate genes may be implicated [188,189]. This decision-making process is summarized in Figure 5, which illustrates the main diagnostic routes and their escalation according to disease context.

Whole-genome sequencing (WGS) provides the most comprehensive view, including non-coding regions, structural variants, and complex genomic rearrangements [20]. Although currently less common in routine diagnostics due to higher cost and analytic complexity, WGS is increasingly considered when WES remains inconclusive, when structural variants are suspected, or in research-oriented contexts aimed at novel variant discovery [23,190]. Variants in non-coding sequences, which escape WES analysis, are often associated with gene dysfunction, and in the case of negative WES/WGS remain a very useful system [191,192].

For hereditary cancer syndromes, comprehensive multigene panels covering both high- and moderate-penetrance genes (e.g., *BRCA1/2*, *MMR* genes) are recommended as first-line tests, as they directly inform genetic counselling, risk reduction strategies, and therapeutic decision-making. Negative or inconclusive results in this setting may justify WES or WGS in selected patients, particularly in research or translational contexts. It should be noted that, depending on the resources and clinical setting, WES and WGS can be considered either as sequential steps (with WGS following a negative WES) or as alternative approaches, with WGS directly chosen in place of WES when maximal variant discovery is required.

Across all scenarios, testing strategies should align with national guidelines and professional society recommendations, while also considering laboratory availability, reimbursement policies, and the clinical utility of the expected results. In summary, clinicians should prioritize targeted panels when the causal genes are well defined, escalate to WES for broader but still coding-focused exploration, and consider WGS for unresolved or complex cases where maximal variant discovery is required.

## 4. Limitations and Future Perspectives

### 4.1. Limitations of Current Approaches

Despite their widespread clinical use, each NGS approach has important limitations.

Targeted NGS gene panels have become invaluable tools in clinical diagnostics due to their cost-effectiveness, relatively rapid turnaround times, and focused analysis of clinically relevant genes [193]. However, these panels have inherent limitations that can impact diagnostic yield and clinical utility. Firstly, targeted panels typically focus on coding regions and canonical splice sites of predefined gene sets. As a consequence, structural variants such as large deletions, duplications, inversions, or complex rearrangements may be underdetected or missed entirely unless complemented by additional techniques like multiplex ligation-dependent probe amplification (MLPA) or array comparative genomic hybridisation (aCGH) [194]. Furthermore, deep intronic variants and regulatory region mutations that affect gene expression or splicing outside the captured regions are generally not interrogated by conventional panels, potentially leaving some pathogenic variants undetected [44]. Secondly, the limited gene content of panels means mutations in novel or less well-characterised genes may be overlooked, especially in genetically heterogeneous disorders or atypical phenotypes. As gene discovery rapidly evolves, static panels risk becoming outdated, necessitating regular updates to maintain clinical relevance [9].

To overcome these challenges, WES and WGS provide broader and more comprehensive variant detection, including noncoding regions and novel genes [195]. Nevertheless, these approaches require more complex bioinformatic analysis, increased data storage, and generate a higher number of variants of uncertain significance (VUS), complicating interpretation and clinical decision-making [19]. WES provides broader coverage of coding regions but suffers from uneven capture efficiency, incomplete exon representation, and limited power to detect copy number or structural variants, while excluding non-coding regions altogether. On the other hand, WGS offers the most comprehensive analysis, capturing both coding and non-coding regions and enabling detection of diverse variant types, yet it remains costly, generates vast and complex datasets, and increases the likelihood of incidental findings, with typically lower sequencing depth than targeted approaches. These inherent challenges highlight the need for complementary strategies and novel technologies to bridge current diagnostic gaps.

### 4.2. Emerging Strategies: Virtual Panels, Phenotype-Driven Tools and AI

Looking forward, the integration of dynamic gene panels, continuously updated through automated pipelines incorporating latest gene–disease associations, will improve diagnostic accuracy [196]. A subset of genes can be extracted from WES or WGS data and analysed as if it were a targeted gene panel. This approach is often referred to as a virtual panel. As documented, virtual panels offer a superior yield compared to static panels [197]. After sequencing the entire exome or genome, bioinformatic filters are applied to focus only on genes of known clinical relevance for a specific condition, such as cardiomyopathies, neurodevelopmental disorders, or dementia. While conventional targeted panels remain more cost-effective and generally provide higher coverage and data quality for a predefined set of genes, virtual panels extracted from WES/WGS add unique value. They enable dynamic reanalysis as novel disease-associated genes are discovered, without the need to resequence the patient, and allow the generation of multiple virtual panels from a single sequencing experiment, thereby increasing flexibility and long-term clinical utility. In this way, virtual panels bridge the gap between focused targeted testing and comprehensive WES/WGS, offering a balance between diagnostic precision, adaptability, and future-proofing of genomic data [198].

Moreover, computational tools that integrate detailed clinical phenotypes with genotype data can substantially improve variant prioritisation and interpretation. These phenotype-driven prioritisation algorithms typically rely on structured phenotypic descriptions, such as those provided by the Human Phenotype Ontology (HPO), and combine them with genomic data to rank variants according to their likelihood of explaining the observed clinical features [199]. Tools such as Exomiser or PhenIX have already demonstrated their utility in clinical diagnostics by reducing the number of candidate variants and prioritising those with the highest clinical relevance. This approach is particularly valuable in genetically heterogeneous conditions, such as neurodevelopmental disorders or cardiomyopathies [200,201]. Recent advances in machine learning (ML) and artificial intelligence (AI) further enhance variant interpretation. For instance, the SEQ platform uses advanced algorithms to classify and prioritize genetic variants based on patient-specific clinical information, such as suspected conditions, observed phenotypes, and age of onset. In one study, 99.89% of variants detected in a whole-exome sequencing (WES) sample were classified at levels IV or V, indicating high clinical relevance [202]. AI-assisted approaches can (i) predict the pathogenicity of novel variants by learning from large sets of known benign and pathogenic variants, (ii) integrate genomic and structured clinical data to prioritise the most likely disease-causing variants, (iii) identify patterns across multiple datasets to help classify variants of uncertain significance (VUS), and (iv) accelerate the time from sequencing to actionable clinical interpretation. Examples of AI-based tools include DeepVariant for variant calling, SpliceAI for splice-site prediction, and EVE (Evolutionary model of Variant Effect) for variant pathogenicity prediction, illustrating how AI leverages large-scale genomic and clinical datasets to improve diagnostic yield and efficiency [203,204,205,206,207].

By combining the flexibility of virtual panels with sophisticated phenotype-driven and AI-assisted approaches, these emerging strategies not only improve diagnostic yield but also support a more precise and efficient translation of genomic data into clinical decision-making.

### 4.3. Long-Read Sequencing and Future Outlook

**Figure 6:**
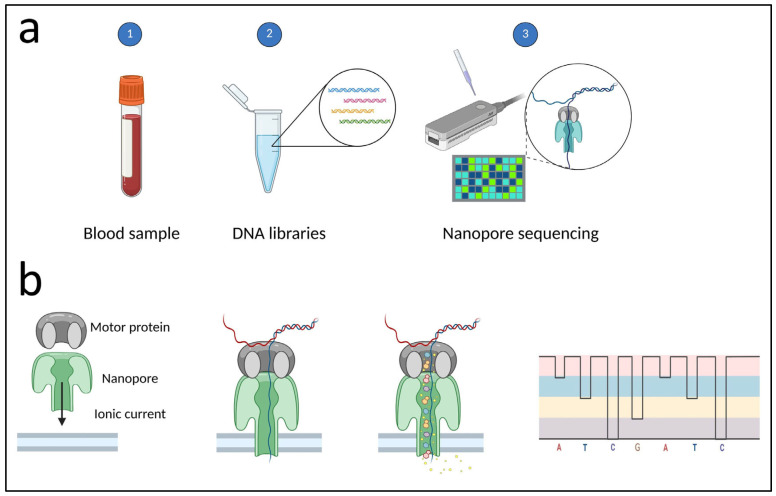
Workflow and principle of nanopore sequencing. (**a**) Schematic overview of the sequencing process, beginning with a blood sample, followed by the preparation of DNA libraries, and subsequent sequencing using a nanopore device. (**b**) During sequencing, motor proteins guide nucleic acids through the nanopore, where each base alters the ionic current in a characteristic manner. These current disruptions are recorded and analysed by a computer, which converts the electrical signals into nucleotide sequences in real time. This technology enables long-read sequencing and the detection of a broad spectrum of genomic variants. The figure was created with BioRender version June 2025 (https://www.biorender.com/) and adapted from a previous work [209]. Created in BioRender. Treccarichi, S. (2025).

As emphasized by several authors, long-read sequencing technologies, such as those developed by PacBio and Oxford Nanopore, are increasingly being explored for clinical diagnostics [208]. Figure 6 briefly illustrates the nanopore workflow used for DNA sequencing.

Unlike short-read approaches, long reads can span several kilobases, enabling the accurate detection of structural variants, highly repetitive or non-unique genomic regions, complex rearrangements, and long-range haplotype phasing, which are often missed by conventional NGS. Their ability to sequence through repetitive or GC-rich regions also improves coverage of previously inaccessible genomic loci. Although still limited by higher costs, lower throughput, and evolving bioinformatics pipelines, long-read sequencing holds great promise for applications in rare disease diagnostics, cancer genomics, and the characterization of neurodevelopmental and neuromuscular disorders [210,211,212]. As technologies mature, their integration into routine clinical practice is expected to complement and, in some contexts, surpass traditional short-read sequencing.

Currently, the clinical applications of long-read sequencing have mainly been explored in research settings. Notably, long-read whole-genome sequencing has demonstrated its potential as a diagnostic tool for genetically unsolved dystrophinopathies, enabling the accurate identification of complex structural variants in the *DMD* gene [213]. Long-read sequencing with the PacBio platform was applied in the Genomic Answers for Kids program, a large cohort of undiagnosed pediatric patients. Despite prior negative results from WES, WGS, microarray, and targeted panels, diagnostic yields ranged from 11% in previously tested patients to 34.5% in genetically naïve patients. Structural variant analysis contributed up to 13% of new diagnoses, although variants and genes of uncertain significance remained the most common finding (58%) [214]. In this context, several studies have integrated long-read sequencing with WES data in cases of autosomal recessive disease where only a single pathogenic variant was detected. Long-read sequencing enables the identification of structural variants, thereby accounting for a fraction of cases in which the second pathogenic allele is not captured by WES [215].

Beyond sequencing technologies, multi-omics approaches are increasingly being applied to improve diagnostic yield in rare diseases. At present, they are most widely applied in oncology, where the complexity and heterogeneity of cancer benefit these approaches [216,217,218]. Metabolic disorders also represent an important target for multi-omics strategies. In one large study, the five most frequently diagnosed conditions were Gaucher disease, Niemann–Pick disease type A/B, phenylketonuria, mucopolysaccharidosis type I, and Wilson disease [219]. By integrating NGS using a 206-gene panel with biochemical analyses, this multi-omics approach achieved a diagnostic yield of 37% in a cohort of 3720 patients.

By integrating genomics with transcriptomics, proteomics, metabolomics, or epigenomics, these strategies provide complementary layers of information that can uncover pathogenic mechanisms not evident from DNA sequencing alone. For example, RNA sequencing can reveal splicing defects or expression outliers, while metabolomic and proteomic profiling may highlight downstream biochemical alterations. Combining these data with genomic findings enhances variant interpretation, facilitates the discovery of novel disease genes, and supports precision medicine strategies.

In summary, while targeted NGS panels remain foundational in molecular diagnostics, their limitations underscore the need for complementary strategies and technological innovations to maximise diagnostic yield and precision medicine implementation.

## 5. Conclusions

NGS approaches have significantly transformed clinical diagnostics by enabling rapid, efficient, and cost-effective identification of pathogenic variants in a wide range of Mendelian disorders [1]. Among them, gene panels are highly applicable in well-defined phenotypes and facilitate streamlined interpretation, often resulting in timely and actionable diagnoses [19]. However, no single approach is sufficient in all clinical contexts. The integration of broader technologies such as WES and WGS is increasingly essential in patients with complex, atypical, or overlapping phenotypes where targeted panels fall short [9,195]. An adaptable, tiered diagnostic strategy—starting from disease-specific panels and escalating to WES or WGS when needed—may offer the optimal balance between diagnostic efficiency and comprehensiveness [196]. As genomics continues to advance, the convergence of panel-based diagnostics, exome-wide approaches, and intelligent data integration will be key to unlocking the full potential of precision medicine [203]. Within this context, long-read sequencing holds great promise for the future of clinical diagnostics, offering the ability to resolve complex variants and expand our understanding of genetic diseases.

In this review, we have provided a comprehensive overview of the different NGS approaches, with a focus on their clinical applications, while underscoring both their advantages and current limitations. Ultimately, the integration of these evolving sequencing strategies into routine diagnostics will accelerate precise and equitable care for patients with rare and common genetic disorders alike.

## Figures and Tables

**Table 1:** Comprehensive comparison of NGS approaches—targeted gene panels, WES, and WGS—as discussed in this review.

Feature	Targeted Gene Panels	WES	WGS
Analyzed region	50–500 selected genes	All coding exons (~1–2% of genome)	Entire genome (coding + non-coding)
Average coverage (depth)	500–1000×	80–150×	30–50×
Average number of mapped reads	5–20 million	50–100 million	600–900 million
Coverage uniformity	Very high (targeted)	Variable (depends on capture efficiency)	High and uniform
Sensitivity for low-frequency variants	High (ideal for mosaicism or VAF < 10%)	Moderate	Lower unless sequenced at high depth
Risk of incidental findings	Low	Moderate	High
Mosaicism detection	Excellent (due to high coverage)	Moderate (depends on depth and region)	Limited at standard coverage; better with >60×
Detection of CNVs/structural variants	Limited	Partial (depends on pipeline)	Excellent
Analysis turnaround time	Fast	Moderate	Slow
Average cost	Low	Moderate	High
Primary clinical indications	Conditions with clear phenotype and known genes	Rare diseases, neuropsychiatric disorders, complex phenotypes	Unresolved cases, complex/multifactorial diseases
Potential for novel gene discovery	None	Moderate	High
Data management burden	Low	Moderate	High (large data volume)

WES, whole-exome sequencing; WGS, whole-genome sequencing; CNVs, copy number variations; VAF, variant allele frequency.

**Table 2:** Key phases and tools in the NGS diagnostic workflow.

Workflow Phase	Description	Technologies/Tools	Critical Aspects
Sample Preparation	DNA extraction and quantification	Qubit, Nanodrop, PCR	DNA integrity, purity, contamination
Library Preparation	DNA fragmentation and adapter ligation	Enzymatic kits, sonicators	Bias in representation, ligation efficiency
Target Enrichment	Capture or amplification of regions of interest (ROI)	Agilent SureSelect, Haloplex	Uniformity, off-target effects
Sequencing	High-throughput parallel sequencing	Illumina, Ion Torrent	Read depth, sequencing errors
Bioinformatics	Alignment, variant calling, annotation	BWA, GATK, ANNOVAR	Pipelines, thresholds, filtering strategy
Interpretation	Clinical classification and reporting	ACMG guidelines	VUS management, evidence strength

BWA: Burrows–Wheeler Aligner; GATK: Genome Analysis Toolkit; ACMG: American College of Medical Genetics and Genomics; VUS: Variant of Uncertain Significance.

**Table 3:** Summarization of the ACMG criteria used for variant classification. This table was adapted from a previous work [19].

Evidence	Criteria Summary
Very Strong (PVS1)	Predicted loss-of-function (LoF) variant in a gene with established LoF disease mechanism (e.g., nonsense, frameshift, canonical ±1 or 2 splice sites, initiation codon loss, large deletions). Use caution with uncertain LoF mechanisms or exon skipping.
Strong (PS1–PS4)	PS1: Same amino acid change as a known pathogenic variant, but caused by a different nucleotide change. PS2: De novo variant (with confirmed maternity and paternity) in a patient with the disease and no family history. PS3: Functional studies support a damaging effect on the gene or protein. PS4: Increased prevalence of the variant in affected individuals vs. controls.
Moderate (PM1–PM6)	PM1: Located in a mutational hotspot or critical functional domain. PM2: Absent or rare in population databases. PM3: Detected in trans (compound heterozygous) with a known pathogenic variant in recessive disease. PM4: Protein length changes (in-frame indels or stop-loss variants). PM5: Missense change at same amino acid as another known pathogenic missense variant. PM6: Assumed de novo (without confirmed maternity/paternity).
Supporting (PP1–PP5)	PP1: Cosegregation with disease in multiple affected family members. PP2: Missense variant in gene with low rate of benign variation and known disease mechanism. PP3: Multiple computational tools predict deleterious effect. PP4: Patient’s phenotype/family history is highly specific to the gene. PP5: Previously reported as pathogenic by reputable source (without primary evidence).

**Table 4:** Variant type detection by NGS technologies.

Variant Type	Targeted Gene Panels	WES	WGS	Main Limitations
SNVs	High sensitivity	High sensitivity	High sensitivity	High sensitivity overall; may be affected by low coverage regions
Indels	≤50 bp	≤50 bp	Up to larger indels	May miss complex/longer indels
CNVs	Known or large CNVs	Variable (coverage/tool-dependent)	Genome-wide	Suboptimal in targeted/WES
SVs	Not detected	Not detected	Detectable (algorithms/depth required)	Needs high-quality data + dedicated tools
Intronic/Regulatory	Not covered	Near-exon only	Genome-wide	Not assessed in targeted/WES
Repeat Expansions	Only if specifically targeted	Low sensitivity	Better, but still challenging	Limitations across all platforms

WES: Whole Exome Sequencing; WGS: Whole Genome Sequencing; SNVs: Single Nucleotide Variants; Indels: small insertions/deletions; CNVs: Copy Number Variants; SVs: Structural Variants. In this table, CNVs refer to copy number changes (deletions/duplications), while SVs refer to more complex rearrangements (inversions, translocations, complex duplications).

**Table 5:** Disease category detection by NGS panels.

Disease Category	Example Disorders	Key Genes	Available Panels
Monogenic Disorders	Cystic fibrosis, Duchenne MD	*CFTR*, *DMD*	Panels target full gene
Neurological Disorders	Huntington, Charcot-Marie-Tooth	*HTT*, *PMP22*, *GJB1*	Often combined with CNV tools
Cardiovascular Disorders	HCM, DCM, ACM	*MYH7*, *TTN*, *LMNA*, *PKP2*	Covered in cardiomyopathy panels
Cancer Syndromes	BRCA-related, Lynch syndrome	*BRCA1*, *BRCA2*, *MLH1*, *MSH2*	Some panels cover >100 genes
Metabolic Disorders	PKU, Gaucher disease	*PAH*, *GBA*	Often phenotype-driven
Intellectual Disabilities	Rett syndrome, Fragile X, ASD	*MECP2*, *FMR1*, *SHANK3*, *SCN2A*	Broad panels often include >500 genes
Mitochondrial Disorders	MELAS, Leigh syndrome	*MT-ND* genes, *POLG*, *SURF1*	Panels may include both nuclear and mtDNA genes
Rare and Undiagnosed Conditions	Atypical syndromes, variable presentations	Varies widely; e.g., *TCF4*, *NRXN1*, *KAT6B*	Ultra-rare disease panels or exome-based panels
Pharmacogenetics (a)	Drug metabolism response	*CYP2D6*, *CYP2C19*, *TPMT*, *SLCO1B1*	Targeted pharmacogenetics panels

MD: Muscular Dystrophy; HCM: Hypertrophic Cardiomyopathy; DCM: Dilated Cardiomyopathy; ACM: Arrhythmogenic Cardiomyopathy; PKU: Phenylketonuria; ASD: Autism Spectrum Disorder; MELAS: Mitochondrial Encephalomyopathy, Lactic Acidosis and Stroke-like episodes; BRCA-related: Hereditary breast and ovarian cancers due to *BRCA1/2* mutations. CNV: Copy Number Variation. *MT-ND* genes: mitochondrial NADH dehydrogenase subunit genes. (a) Drug response and toxicity: these panels are often used to guide therapeutic decisions rather than for diagnostic purposes.

**Table 6:** Genes involved in *cardiomyopathies*: genomic features (from GRCh38/hg38).

Gene	Cardiomyopathies	RefSeq No.	Chr. band	Chr. Position (a)	Size, bp	Exons	MOI
*ACTC1* *	HCM, RCM/LVNC	NM_005159.5	15q14	34,790,230	5320	7	AD
*BAG3* *	DCM	NM_004281.4	10q26.11	119,651,380	26,440	4	AD
*DSC2*	ACM	NM_024422.6	18q12.1	31,058,840	43,582	16	AR, AD
*DSG2*	ACM	NM_001943.5	18q12.1	31,498,177	50,832	15	AD
*DSP* *	DCM	NM_004415.4	6p24.3	7,541,671	45,044	24	AD, AR
*FLNC* *	DCM, ACM, RCM/LVNC	NM_001458.5	7q32.1	128,830,406	28,867	48	AD
*GLA*	HCM	NM_000169.3	Xq22.1	101,397,803	10,123	7	X-linked
*JUP*	ACM	NM_002230.4	17q21.2	41,754,609	32,103	14	AR, AD
*LAMP2*	HCM	NM_002294.3	Xq24	120,426,148	43,149	9	X-linked
*LMNA* *	DCM, ACM	NM_170707.4	1q22	156,114,711	25,371	12	AD
*MYBPC3* *	HCM	NM_000256.3	11p11.2	47,331,406	21,297	35	AD
*MYH7* *	DCM, HCM, RCM/LVNC	NM_000257.4	14q11.2	23,412,740	22,921	40	AD
*PKP2* *	ACM	NM_001407159.1	12p11.21	32,790,755	106,023	13	AD
*PRKAG2*	HCM	NM_016203.4	7q36.1	151,556,127	320,989	16	AD
*RBM20* *	DCM	NM_001134363.3	10q25.2	110,644,336	195,133	14	AD
*SCN5A* *	DCM	NM_000335.5	3p22.2	38,548,062	101,626	28	AD
*TMEM43*	ACM	NM_024334.3	3p25.1	14,125,052	18,629	12	AD
*TNNI3*	HCM, RCM/LVNC	NM_000363.5	19q13.42	55,151,767	5966	8	AD
*TNNT2*	HCM	NM_001276345.2	1q32.1	201,359,014	18,667	17	AD
*TPM1*	HCM	NM_001018005.2	15q22.2	63,042,747	23,432	10	AD
*TTN* *	DCM, RCM/LVNC	NM_001267550.2	2q31.2	178,525,989	281,435	363	AD, AR (b)

HCM: hypertrophic cardiomyopathy; DCM: Dilated Cardiomyopathy; ACM: Arrhythmogenic Cardiomyopathy; RCM: Restrictive Cardiomyopathy; LVNC: Left Ventricular Noncompaction; Chr: Chromosome; RefSeq: Reference Sequence; MOI: Mode of Inheritance; AD: Autosomal Dominant; AR: Autosomal Recessive; * Genes with well-established diagnostic relevance and high clinical impact in inherited cardiomyopathies, commonly included in first-tier NGS panels. (a) Genomic coordinates from the p-arm telomere. (b) Autosomal recessive inheritance is rarely reported for *TTN*-related cardiomyopathies and its clinical interpretation may be challenging.

**Table 7:** List of key genes originally incorporated in the MRC Dementia Gene Panel.

Gene (1)	RefSeq	Chr	MOI	ND	OMIM ID
*PRNP*	NM_000311	20p13	ADo	Prion disease	176640
*PSEN1*	NM_000021	14q24.2	ADo	Early-onset AD	104311
*PSEN2*	NM_000447	1q42.13	ADo	Early-onset AD	600759
*APP*	NM_000484	21q21.3	ADo	Early-onset AD	104760
*GRN*	NM_002087	17q21.31	ADo	Frontotemporal dementia	138945
*MAPT*	NM_005910	17q21.31	ADo	FTD, PSP, PPND	157140
*TREM2*	NM_018965	6p21.1	AR; H	Nasu–Hakola; H: risk for AD/FTD	605086
*CHMP2B*	NM_014043	3p11.2	ADo	FTD-3	609215
*CSF1R*	NM_005211	5q32	ADo	Adult-onset leukoencephalopathy	164770
*FUS*	NM_004960	16p11.2	ADo	ALS, FTD overlap	137070
*ITM2B*	NM_004534	13q14.2	Ado	Familial Danish/Belgian dementia	605637
*NOTCH3*	NM_000435	19p13.12	ADo	CADASIL	600276
*SERPINI1*	NM_000605	3q26.1	Ado	Familial encephalopathy with neuroserpin inclusion bodies	602445
*TARDBP*	NM_007375	1p36.22	ADo	ALS, FTD overlap	605078
*TYROBP*	NM_003332	19q13.12	AR; H	Nasu–Hakola disease; H: risk in dementia	604195
*VCP*	NM_007126	9p13.3	ADo	IBMPFD, ALS, FTD	601023

(1) from [181]. RefSeq, transcript ID; Chr, chromosomal location; MOI, mode of inheritance; ND, associated neurodegenerative conditions; ADo, autosomal dominant; AR, autosomal recessive; H, heterozygous. AD, Alzheimer’s disease; FTD, Frontotemporal Dementia; PSP, Progressive Supranuclear Palsy; PPND, Pallido-Ponto-Nigral Degeneration; ALS, Amyotrophic Lateral Sclerosis; CADASIL, Cerebral Autosomal Dominant Arteriopathy with Subcortical Infarcts and Leukoencephalopathy; IBMPFD, Inclusion Body Myopathy with Paget Disease of Bone and Frontotemporal Dementia.

## Data Availability

No new data were created or analyzed in this study. Data sharing is not applicable to this article.

## Abbreviations

The following abbreviations are used in this manuscript:

ACM	Arrhythmogenic Cardiomyopathy
ACMG	American College of Medical Genetics and Genomics
ACTA2	Actin Alpha 2, Smooth Muscle
ACTC1	Actin Alpha Cardiac Muscle 1
AD	Alzheimer’s Disease
ALS	Amyotrophic Lateral Sclerosis
AMP	Association for Molecular Pathology
AOS	Aneurysm–Osteoarthritis Syndrome
APOB	Apolipoprotein B
ARVC	Arrhythmogenic Right Ventricular Cardiomyopathy
ASD	Autism Spectrum Disorders
BAG3	BAG Cochaperone 3
BCKDHA	Branched Chain Keto Acid Dehydrogenase E1 Subunit Alpha
BCKDHB	Branched Chain Keto Acid Dehydrogenase E1 Subunit Beta
BRCA1	BRCA1 DNA Repair Associated
BRCA2	BRCA2 DNA Repair Associated
BrS	Brugada syndrome
BWA	Burrows–Wheeler Aligner
cbEGF	calcium-binding EGF-like domain
CDG	Congenital Disorder of Glycosylation
CFTR	Cystic Fibrosis Transmembrane Conductance Regulator
CMA	Chromosomal Microarray
CMT	Charcot–Marie–Tooth disease
CNVs	Copy Number Variants
COL3A1	Collagen Type III Alpha 1 Chain
CYP2C19	Cytochrome P450 Family 2 Subfamilt C Member 19
CYP2D6	Cytochrome P450 Family 2 Subfamily D Member 6
DBT	Dihydrolipoamide Branched Chain Transacylase E2
DCM	Dilated Cardiomyopathy
DES	Desmin
DMD	Dystrophin gene
DSC2	Desmocollin 2
DSG2	Desmoglein 2
DSM-5	Diagnostic and Statistical Manual of Mental Disorders, Fifth Edition
DSP	Desmoplakin
FBN1	Fibrillin 1
FFPE	Formalin-Fixed, Paraffin-Embedded
FLNC	Filamin C
FMR1	Fragile X Messenger Ribonucleoprotein 1
FTAAD	Familial Thoracic Aortic Aneurysms And Dissections
GATK	Genome Analysis Toolkit
GBA	Glucosylceramidase Beta 1
GJB1	Gap Junction Protein Beta 1
GLA	Galactosidase Alpha
HCM	Hypertrophic Cardiomyopathy
HGMD	Human Gene Mutation Database
HTT	Huntingtin
ICD	Implantable Cardioverter-Defibrillators
ID	Intellectual Disabilities
IDD	Intellectual and Developmental Disabilities
IEM	Inborn Errors of Metabolism
JUP	Junction Plakoglobin
KAT6B	Lysine Acetyltransferase 6B
KCNQ1	Potassium Voltage-Gated Channel Subfamily Q Member 1
LAMP2	Lysosomal Associated Membrane Protein 2
LBD	Lewy Dody Dementia
LDLR	Low Density Lipoprotein Receptor
LDS	Loeys–Dietz Syndrome
LMNA	Lamin A/C
LoF	Loss-of-function
LOVD	Leiden Open Variation Database
LQTS	Long QT Syndrome
LVH	Left Ventricular Hypertrophy
LVNC	Left Ventricular Noncompaction
MCI	Mild Cognitive Impairment
MDT	Multidisciplinary team meetings
MECP2	Methyl CpG Binding Protein 2
MELAS	Mitochondrial Encephalomyopathy, Lactic Acidosis and Stroke-like episodes
MFS	Marfan Syndrome
MLH1	MutL Homolog 1
MLPA	Multiplex Ligation-dependent Probe Amplification
MSH2	MutS Homolog 2
MSH6	MutS Homolog 6
MSUD	Maple Syrup Urine Disease
mtDNA	mitochondrial DNA
MYBPC3	Myosin Binding Protein C3
MYBPC3	Myosin-Binding Protein C3
MYH11	Myosin Heavy Chain 11
MYH7	Myosin Heavy Chain 7
MYLK	Myosin Light Chain Kinase
NDDs	Neurodevelopmental disorders
NGS	Next-generation sequencing
NRXN1	Neurexin 1
PAH	Phenylalanine Hydroxylase
PCSK9	Proprotein Convertase Subtilisin/Kexin Type 9
PD	Parkinson’s Disease
PKP2	Plakophilin 2
PKU	Phenylketonuria
PMP22	Peripheral Myelin Protein 2
PMS2	PMS1 Homolog 2, Mismatch Repair System Component
POLG	Mitochondrial Polymerase Gamma Catalytic Subunit
PRKAG2	Protein Kinase AMP-Activated Non-Catalytic Subunit Gamma 2
PRKG1	Protein kinase CGMP-Dependent 1
RBM20	RNA Binding Motif Protein 20
RCM	Restrictive Cardiomyopathy
RYR2	Ryanodine Receptor 2
SCD	Sudden Cardiac Death
SCN2A	Sodium Voltage-Gated Channel Alpha Subunit 2
SCN5A	Sodium Voltage-Gated Channel Alpha Subunit 5
SHANK3	SH3 And Multiple Ankyrin Repeat Domains 3
SLCO1B1	Solute Carrier Organic Anion Transporter Family Member 1B1
SMA	Spinal muscular atrophy
SMAD3	SMAD Family Member 3
SNV	Single Nucleotide Variant
STR	Short Tandem Repeat
SURF1	Surfeit Locus Protein 1
TCF4	Transcription Factor 4
TGF-β	Transforming Growth Factor-Beta
TGFBR1	Transforming Growth Factor Beta Receptor 1
TGFBR2	Transforming Growth Factor Beta Receptor 2
TMEM43	Transmembrane Protein 43
TNNI3	Troponin I3, Cardiac Type
TNNT2	troponin T2, Cardiac Type
TPM1	Tropomyosin 1
TPMT	Thiopurine S-Methyltransferase
TTN	Titin
UMI	Unique Molecular Identifiers
VCF	VCF Variant Call Format
vEDS	vascular Ehlers–Danlos Syndrome
VUS	Variants of Uncertain Significance
